# Experimental and Modeling Evaluation of Dimethoxymethane as an Additive for High-Pressure Acetylene Oxidation

**DOI:** 10.1021/acs.jpca.2c03130

**Published:** 2022-09-01

**Authors:** Lorena Marrodán, Ángela Millera, Rafael Bilbao, María U. Alzueta

**Affiliations:** Aragón Institute of Engineering Research (I3A), Department of Chemical and Environmental Engineering, University of Zaragoza, R+D building, Río Ebro Campus, 50018 Zaragoza, Spain

## Abstract

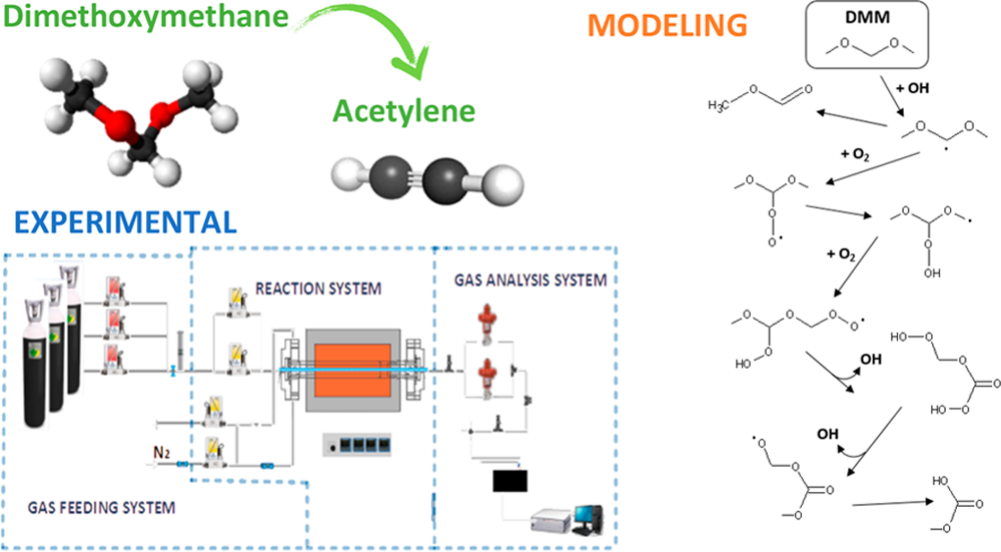

The high-pressure oxidation of acetylene–dimethoxymethane (C_2_H_2_–DMM) mixtures in a tubular flow reactor has been analyzed from both experimental and modeling perspectives. In addition to pressure (20, 40, and 60 bar), the influence of the oxygen availability (by modifying the air excess ratio, λ) and the presence of DMM (two different concentrations have been tested, 70 and 280 ppm, for a given concentration of C_2_H_2_ of 700 ppm) have also been analyzed. The chemical kinetic mechanism, progressively built by our research group in the last years, has been updated with recent theoretical calculations for DMM and validated against the present results and literature data. Results indicate that, under fuel-lean conditions, adding DMM enhances C_2_H_2_ reactivity by increased radical production through DMM chain branching pathways, more evident for the higher concentration of DMM. H-abstraction reactions with OH radicals as the main abstracting species to form dimethoxymethyl (CH_3_OCHOCH_3_) and methoxymethoxymethyl (CH_3_OCH_2_OCH_2_) radicals are the main DMM consumption routes, with the first one being slightly favored. There is a competition between β-scission and O_2_-addition reactions in the consumption of both radicals that depends on the oxygen availability. As the O_2_ concentration in the reactant mixture is increased, the O_2_-addition reactions become more relevant. The effect of the addition of several oxygenates, such as ethanol, dimethyl ether (DME), or DMM, on C_2_H_2_ high-pressure oxidation has been compared. Results indicate that ethanol has almost no effect, whereas the addition of an ether, DME or DMM, shifts the conversion of C_2_H_2_ to lower temperatures.

## 1 Introduction

It is well-known that the addition of oxygenates to diesel may have beneficial effects in terms of exhaust emissions.^1,2^ The higher oxygen content of these compounds results in a cleaner combustion leading to reduced diesel engine emissions, especially soot. An explanation to this fact can be found in a decrease of C–C bonds in favor of C–O bonds. A polyether, such as the family of poly(oxymethylene) dimethyl ethers (POMDMEs) or oxymethylene ethers (OMEs), with a molecular structure of CH_3_–O–(CH_2_–O)_*n*_–CH_3_, should be an efficient additive. These compounds have attracted a lot of attention because of their generally high cetane number and oxygen content, the absence of C–C bonds that allows an almost soot-free combustion, as well as low NO_*x*_ emissions.^3−5^ The presence of methylene groups attached to oxygen atoms in the structure of the OMEs leads to the formation of hydroperoxides in the early stages of the combustion. These peroxides react through complex mechanisms that include O_2_ additions and several isomerizations and decompositions during which highly reactive OH radicals are generated. These OH radicals subsequently degrade soot precursors by oxidative processes.^6,7^

The POMDME with *n* = 0, dimethyl ether (DME, CH_3_–O–CH_3_), is well-known for its high reactivity at low temperatures and the hydroperoxide reaction mechanism responsible for its characteristic negative temperature coefficient (NTC) zone. The DME oxidation chemistry has been extensively analyzed as summarized by Rodriguez et al.,^8^ who reported 34 different experimental studies carried out under a wide range of operational conditions and devices. Experimental studies show that blends of DME and diesel, depending on the operating conditions, can reduce emissions of smoke, NO_*x*_, carbon monoxide, and unburned hydrocarbons.^9^ However, the use of DME as a diesel fuel additive can have some disadvantages such as an increase in the vapor pressure, a decrease in the fuel viscosity, and lower solubility at low temperatures,^10,11^ as well as a reduction in the lower calorific value,^12^ that will imply several engine modifications^13^

As *n* increases, properties such as the cetane number improve. In comparison to DME, dimethoxymethane (DMM, CH_3_–O–CH_2_–O–CH_3_), with chain length *n* = 1, has a higher quantity of oxygen, lower vapor pressure, and better solubility with diesel fuel. A remarkable reduction in CO and smoke emissions^14^ as well as an improvement in thermal efficiency^15^ can be achieved when operating with diesel-DMM blends. The combustion kinetics of DMM has been previously analyzed in terms of experimental studies,^7,16−26^ chemical kinetic modeling,^7,16,18,20−24,26^ and theoretical calculations.^23,24,27^

The oxidation of mixtures of hydrocarbons and DMM has been previously tested in the literature, mainly in flames. Renard et al.^28^ observed a reduction in the maximum mole fraction of the intermediate species identified as soot precursors due to the addition of DMM to premixed ethylene/oxygen/argon flames. Sinha and Thomson^17^ suggested that the addition of DMM to propene opposed flow diffusion flames reduces the formation of ethylene, acetylene and propylene due to the lack of C–C bonds. During their study of the effect of DMM addition to premixed *n*-heptane flames, Chen et al.^29^ found that the concentration of the experimentally quantified C_1_–C_5_ intermediates was reduced. To our knowledge, there is a lack of studies in the literature that analyze the effects of DMM addition on the oxidation of hydrocarbons, performed in experimental devices other than flames.

In this context, the aim of the present work is (i) to conduct high-pressure experiments of acetylene (C_2_H_2_) and DMM mixtures in a tubular flow reactor and carefully controlled conditions, which will extend the existing database; C_2_H_2_ has been selected as it is recognized as a soot precursor;^30^ (ii) to update our chemical kinetic mechanism with recent theoretical calculations. Therefore, the present work brings new experimental data on the oxidation regimen of DMM, the simplest member of the POMDMEs family which includes promising fuel additives.

In addition, the influence of the addition of different oxygenates proposed as prospective additives on the oxidation of C_2_H_2_ will be analyzed. Therefore, results obtained during the high-pressure oxidation of C_2_H_2_–ethanol/DME/DMM mixtures, in the same experimental setup,^31,32^ will be compared.

## 2 Methods

### 2.1 Experimental Section

The experiments have been performed in a tubular flow reactor included in a setup that has been previously used and described in earlier works of the research group on high-pressure oxidation (e.g., refs (20), (33)). Therefore, only the most important features will be highlighted here.

Table 1 details the main conditions of the C_2_H_2_–DMM mixtures high-pressure oxidation experiments. Two different DMM concentrations have been tested (70 and 280 ppm, approximately), corresponding, respectively, to 10 and 40% of the inlet C_2_H_2_ concentration (about 700 ppm), which are the lowest and the highest percentage used in previous works on the effect of the addition of oxygenates to C_2_H_2_ performed by our research group, which allows a comparison of the effect of different compounds analyzed.^32,34−36^ These amounts were enough to draw conclusions on the effects of the addition of different oxygenated compounds. Moreover, these percentages (10 and 40% of the fuel concentration) cover the ranges used in other literature studies on the oxidation of DMM–hydrocarbon mixtures, as is the case of the work of Chen et al.^29^ who studied the effect of DMM addition (25% of the inlet HC concentration) to *n*-heptane flames.

**Table 1:** Matrix of Experimental Conditions^a^

set	C_2_H_2_ [ppm]	DMM [ppm]	O_2_ [ppm]	pressure [bar]	λ
1	723	68	1386	20	0.67
2	712	280	2010	20	0.71
3	735	61	2045	20	0.98
4	756	271	3110	20	1.05
5	751	75	45600	20	21.16
6	758	284	59945	20	19.78
7	708	70	1564	40	0.76
8	758	304	2102	40	0.68
9	690	70	2035	40	1.02
10	772	267	3100	40	1.03
11	815	75	46000	40	19.68
12	740	275	62400	40	21.53
13	767	72	1515	60	0.69
14	740	284	2000	60	0.67
15	755	66	2030	60	0.94
16	759	291	2870	60	0.94
17	760	73	45750	60	20.99
18	679	285	58670	60	20.68

a Experiments are conducted in the 450–1050 K temperature range. The balance is closed with N_2_.

Reactants (C_2_H_2_ and DMM) are fed from gas cylinders and diluted in N_2_ to minimize the reaction thermal effects that can take place in a tubular flow reactor designed to approximate plug flow (6 mm inner diameter and 1500 mm total length).^37^ Oxidation experiments have been performed for three different manometric pressures (20, 40, and 60 bar) and in the temperature range of 450–1050 K. The experiments have been carried out for different oxygen concentrations, from fuel-rich to fuel-lean conditions; i.e., three different air excess ratios (λ) have been tested, λ ≈ 0.7, 1 and 20, with λ being the inlet oxygen concentration divided by the stoichiometric, calculated considering both fuel components, acetylene and DMM.

To control and maintain the desired pressure inside the reactor, the setup has a differential pressure transducer controlled by a pneumatic valve situated downstream. The reactor is enclosed in a stainless-steel tube which acts as a pressure shell, and nitrogen gas is delivered to the shell side of the reactor to obtain a similar pressure to that inside. The reactor–pressure shell system is placed inside a three zone electrically heated furnace and K-thermocouples located in the void between the reactor and the shell have been used to measure the longitudinal temperature profiles, resulting in an isothermal (±10 K) reaction zone of 560 mm. For these conditions, and a total gas flow rate of 1 L (STP)/min, the gas residence time within isothermal reaction zone is represented by eq 1.
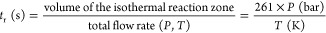
1The experimentally determined temperature profiles inside the reactor for a flow rate of 1 L (STP)/min and 20, 40, and 60 bar have been included in the Supporting Information (Figures S1–S3).

Finally, downstream of the reactor, the pressure is reduced until atmospheric level and gases are analyzed using a micro gas chromatograph (Agilent 3000A) equipped with TCD detectors. The uncertainty of the measurements can be estimated as ±5%. Three different chromatograms have been included in the Supporting Information (Figures S4–S6), one for each module of the gas chromatograph, in which the different compounds that have been identified and calibrated with the corresponding standards can be seen. This configuration allows the quantification of reactants DMM, C_2_H_2_, and several products such as CO, CO_2_, methyl formate (CH_3_OCHO, MF), CH_4_, and CH_2_O. It is also possible to measure C_2_H_4_ and C_2_H_6_, but they have not been detected in appreciable quantities.

### 2.2 Chemical Kinetic Model

The basic mechanism used in this work was able to describe the high-pressure oxidation of previous mixtures of C_2_H_2_–oxygenates, such as ethanol^31^ and DME.^32^

Regarding the compound of interest in this work, the DMM reaction subset was mainly taken from the work on the high-pressure oxidation of DMM in a tubular flow reactor.^20^ That study exposed the existing uncertainty in the chemical kinetic parameters of some reactions. By analogy to the behavior of another POMDME, the DME, during the oxidation of DMM, peroxy species could be formed; therefore, several reactions were included in the DMM subset (more details can be found in ref (20)).

As stated in the Introduction, recent theoretical calculations have been carried out at the CBS-QB3 level of theory and a new kinetic model has been developed and validated by Vermeire et al.^23^ Therefore, the DMM reaction subset, included in the mechanism previously used by our research group,^31,32^ has been revised, updated, and modified accordingly.

The main modifications done in the present work are summarized in Table 2, including those new reactions added or whose kinetic parameters have been modified (source: Vermeire et al.^23^). These modifications involve the definition of new species whose thermodynamic data have been taken from the same source as the kinetic parameters.

**Table 2:** Reactions for DMM Modified or Added from Vermeire et al.^23^ Compared to Marrodán et al.’s Work^20^^a^

reaction	*A*	*n*	*E*_a_
CH_3_OCH_2_OCH_3_ + O_2_ = CH_3_OCH_2_OCH_2_ + HO_2_	1.88 × 10^4^	2.82	42590.82
CH_3_OCH_2_OCH_3_ + O_2_ = CH_3_OCHOCH_3_ + HO_2_	1.26 × 10^7^	1.99	40344.16
CH_3_OCHOCH_3_ = CH_3_OCHO + CH_3_	6.17 × 10^8^	1.29	13647.22
CH_3_OCH_2_OCH_3_ + OH = CH_3_OCH_2_OCH_2_ + H_2_O	2.03 × 10^–1^	4.22	–5712.23
CH_3_OCH_2_OCH_3_ + OH = CH_3_OCHOCH_3_ + H_2_O	1.00 × 10^5^	2.48	–3680.68
CH_3_OCH_2_OCH_3_ + HO_2_ = CH_3_OCH_2_OCH_2_ + H_2_O_2_	1.32 × 10^1^	3.55	12691
CH_3_OCH_2_OCH_3_ + HO_2_ = CH_3_OCHOCH_3_ + H_2_O_2_	2.62 × 10^2^	3.16	11759
CH_3_OCH_2_OCH_3_ + H = CH_3_OCH_2_OCH_2_ + H_2_	5.04 × 10^6^	2.30	6453.15
CH_3_OCH_2_OCH_3_ + H = CH_3_OCHOCH_3_ + H_2_	2.18 × 10^10^	1.15	6548.75
CH_3_OCH_2_OCH_3_ + O = CH_3_OCH_2_OCH_2_ + OH	5.43 × 10^6^	2.14	3080.78
CH_3_OCH_2_OCH_3_ + O = CH_3_OCHOCH_3_ + OH	1.10 × 10^6^	2.45	2820.26
CH_3_OCH_2_OCH_3_ + CH_3_O = CH_3_OCH_2_OCH_2_ + CH_3_OH	9.8 × 10^2^	2.93	3441
CH_3_OCH_2_OCH_3_ + CH_3_O = CH_3_OCHOCH_3_ + CH_3_OH	3.38 × 10^5^	2.12	4493.30
CH_3_OCH_2_OCH_3_ = CH_3_ + CH_3_OCH_2_O	8.50 × 10^41^	–7.95	91802.09
CH_3_OCH_2_OCH_3_ = CH_3_O + CH_3_OCH_2_	1.24 × 10^25^	–2.29	85325.04
CH_3_OCH_2_OCH_2_ = CH_2_O + CH_3_OCH_2_	2.49 × 10^14^	–0.04	24737.09
CH_3_OCH_2_OCH_2_ + O_2_ = CH_3_OCH_2_OCH_2_O_2_	8.9 × 10^10^	0.23	–1577.43
CH_3_OCH_2_OCH_2_O_2_ = CH_3_OCHOCH_2_O_2_H	5.37 × 10^8^	0.76	14651.05
CH_3_OCHOCH_2_O_2_H = HO_2_CH_2_OCHO + CH_3_	4.05 × 10^12^	0.52	15718
CH_3_OCHOCH_2_O_2_H = CH_3_OCHO + CH_2_O + OH	6.77 × 10^11^	0.32	13025.81
C_3_H_7_O_6_r_7 = HOOCH_2_OCOOCH_3_ + OH	2.03 × 10^9^	1.21	37806
CH_3_OCHOCH_3_ + O_2_ = CH_3_OCOOHOCH_3_	1.04 × 10^15^	–0.92	–119.50
CH_3_OCOOHOCH_3_ = CH_2_OCOOH_2_OCH_3_	0.92 × 10^6^	1.53	17238.00
CH_2_OCOOH_2_OCH_3_ + O_2_ = CH_3_OCOOH_2_OCH_2_O_2_	1.03 × 10^11^	0.23	–1577.43
CH_3_OCOOH_2_OCH_2_O_2_ = HOOCH_2_OCOOCH_3_ + OH	2.64 × 10^10^	0.80	17141.00
HOOCH_2_OCOOCH_3_ = OCH_2_OCOOCH_3_ + OH	1.5 × 10^16^	0.00	42853.72
OCH_2_OCOOCH_3_ = HOCOOCH_3_ + HCO	5.12 × 10^10^	0.65	13479.92
CH_3_OCOOHOCH_3_ = C_3_H_7_O_4_r_2	0.92 × 10^6^	1.53	17238
C_3_H_7_O_4_r_2 + O_2_ = C_3_H_7_O_6_r	1.03 × 10^11^	0.23	–1577.43
C_3_H_7_O_6_r = HOOCH_2_OCOOCH_3_ + OH	2.64 × 10^10^	0.806	17141

a Units: cm^3^, mol, s, and cal.

The final mechanism compiled in the present work involves 151 species and contains 804 reactions. It is provided in the Supporting Information along the corresponding thermodynamic data, both as. txt files. Numerical calculations have been conducted with the plug-flow reactor module of the CHEMKIN-PRO software package^38^ and taking into account the temperature profiles experimentally determined (Supporting Information, Figures S1–S3).

**Figure 1:**
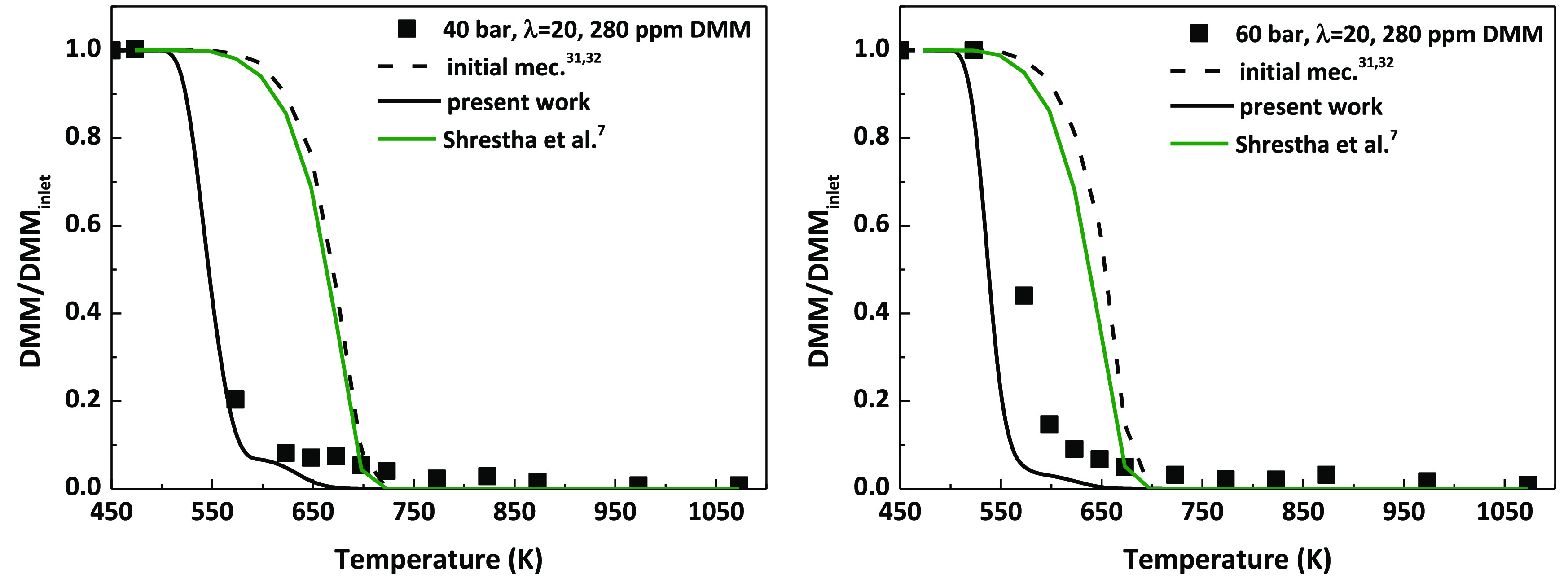
Comparison of the results obtained before (initial mechanism^31,32^) and after the modifications done to the mechanism (present work) for the conditions denoted as sets 12 and 18 in Table 1. Results obtained with Shrestha et al.’s mechanism^7^ for the same conditions are also shown.

The modifications performed to the mechanism have allowed a better match between experimental results and modeling calculations with respect to the starting mechanism (successfully used in previous works of our research group such as refs (31), (32)), especially in the case of fuel-lean conditions and the highest DMM concentration tested. Figure 1 shows an example of the comparison of the results obtained with both mechanisms. Additionally, modeling calculations obtained with a recent DMM chemical kinetic mechanism^7^ have been included in Figure 1 (green lines, for interpretation of the color references, the reader is referred to the web version of the article). The results corroborate the need to continue working on the kinetic mechanism for better prediction of fuel-lean conditions.

First of all, the new mechanism has been evaluated against literature data obtained on different devices and with a wide range of experimental conditions. Specifically, the results obtained by Vermeire et al.^23^ in a jet-stirred reactor (JSR), from pyrolysis to fuel-lean conditions (equivalence ratio values: ø = ∞, ø = 2, ø = 1, and ø = 0.25), have been used to validate the kinetic mechanism, along with tubular flow reactor experimental results reported by Marrodán et al.^21,20^ In the first case,^21^ experiments were conducted at atmospheric pressure from pyrolysis to fuel-lean conditions (i.e., the air excess ratio was varied from λ = 0 to λ = 35), whereas in the second case^20^ the experiments were carried out under high-pressure conditions (20–60 bar) from λ = 0.7 to λ = 20. In addition, the ignition delay times reported by Li et al.,^26^ measured in a shock tube at 1 and 4 atm, have been compared with modeling calculations with the present mechanism.

The different type of reactor and the different pressure range make the selected data set ideal for validation of the new kinetic mechanism at different conditions. The comparison of modeling calculations with the experimental data is given in the Supporting Information, Figures S7–S20. In general, the consumption of DMM and the formation of the main products quantified in the different studies are well caught by the model.

## 3 Results and Discussion

**Figure 2:**
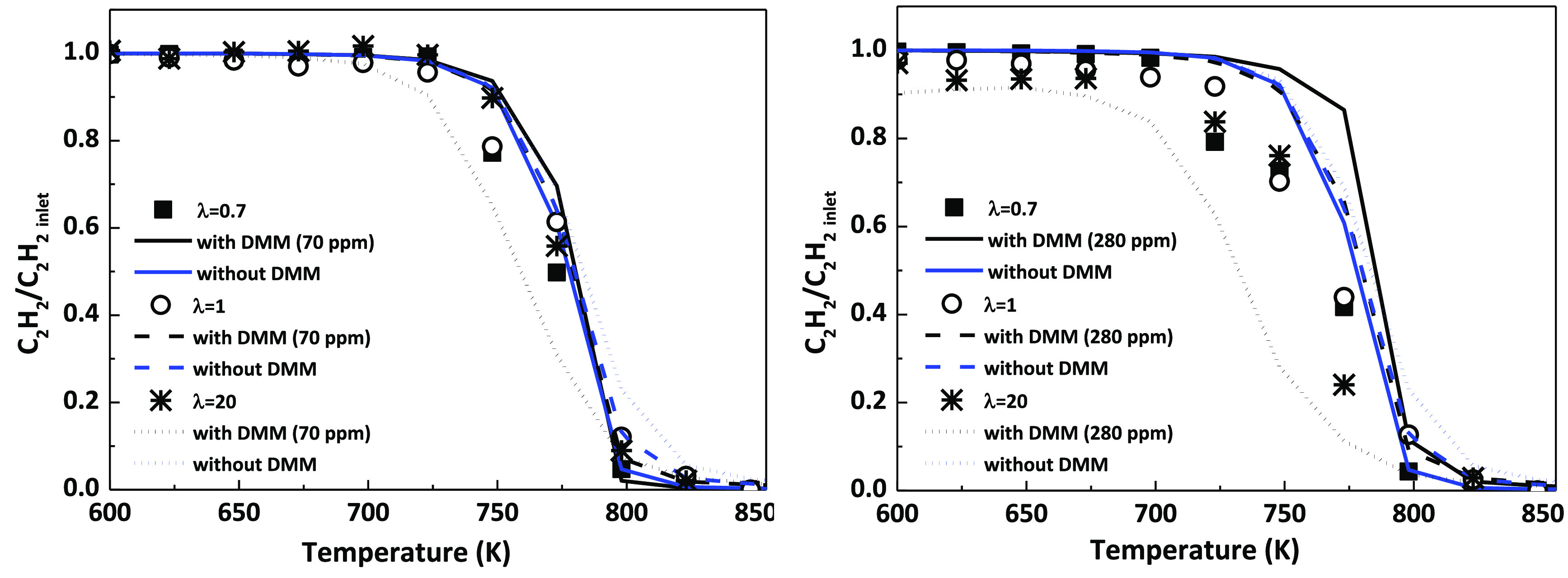
Influence of the addition of DMM on the oxidation of C_2_H_2_ at high pressure (20 bar). Conditions denoted as sets 1–6 in Table 1.

The impact of the presence of DMM on the high-pressure oxidation of C_2_H_2_ has been evaluated for the different air excess ratios (λ) analyzed and the two concentrations of DMM tested (70 and 280 ppm, approximately). Figure 2 shows the results of this evaluation for a pressure of 20 bar. Throughout the paper, experimental results are denoted by symbols and modeling calculations are indicated by lines. For an easier comparison of the results, C_2_H_2_ concentration has been normalized with respect to its inlet concentration (approximately, 700 ppm). In the case of the C_2_H_2_ oxidation in the absence of DMM, only modeling calculations are shown (blue lines, for interpretation of the color references, the reader is referred to the web version of the article), since the present mechanism has been compared with literature data on C_2_H_2_ oxidation at high pressure^39^ showing a good performance (Supporting Information, Figure S21).

As it can be seen, the presence of DMM only modifies the consumption profile of C_2_H_2_ under fuel-lean conditions, shifting its conversion to lower temperatures. The greater the amount of DMM in the reactant mixture, the more emphasized the shift.

**Figure 3:**
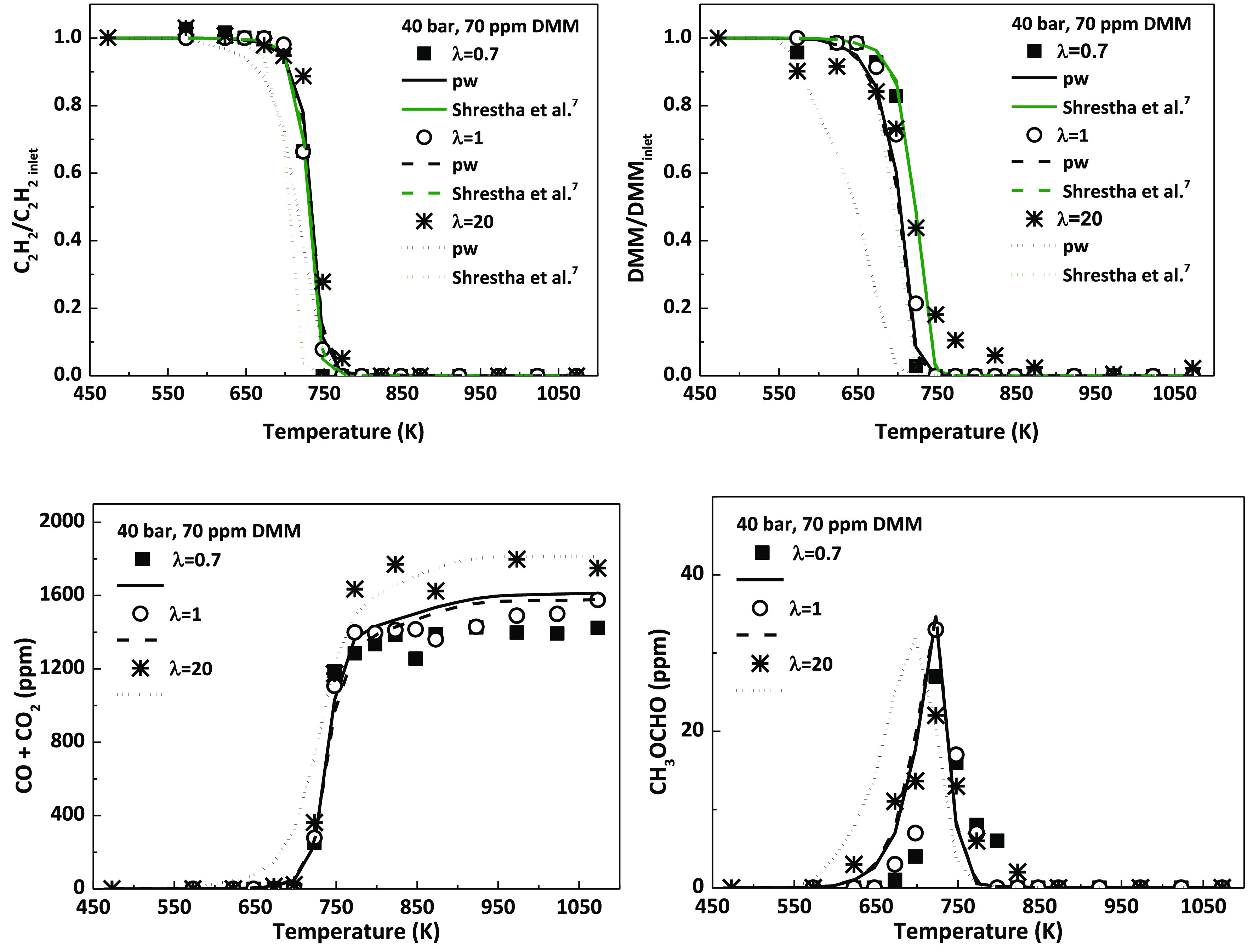
Influence of the air excess ratio (λ) on the concentration profiles of C_2_H_2_, DMM, CO+CO_2_, and CH_3_OCHO (methyl formate) as a function of temperature, for 40 bar and 70 ppm of DMM. Conditions denoted as sets 7, 9, and 11 in Table 1. Results obtained with Shrestha et al.’s mechanism^7^ for C_2_H_2_ and DMM are also shown.

The influence of the oxygen availability in the reactant mixture on the high-pressure oxidation of C_2_H_2_–DMM mixtures has been analyzed. As an example, Figure 3 shows a comparison of the experimental and modeling results obtained for the three different air excess ratios evaluated (λ = 0.7, λ = 1 and λ = 20) for a pressure of 40 bar. The DMM and C_2_H_2_ inlet concentrations have been kept constant at around 70 and 700 ppm, respectively. As previously done, for an easier comparison of the results, DMM and C_2_H_2_ concentrations have been normalized with respect to their inlet concentration, while the concentration of CO and CO_2_, as the main oxidation products quantified, are presented together. Methyl formate (CH_3_OCHO) has been quantified as one of the main intermediate species, and an example of the measured and predicted concentrations is also shown in Figure 3.

From an experimental point of view, there is almost no influence of the air excess ratio (λ) on the consumption of the reactants and products formation. The largest discrepancy between experimental data and modeling calculations is obtained in the case of fuel-lean conditions, when model results are slightly ahead of the experimental data. This fact is due to the modifications made to the mechanism, such as the inclusion of reactions involving the formation of peroxy species from both DMM radicals, CH_3_OCHOCH_3_ and CH_3_OCH_2_OCH_2_, and their subsequent conversion, which are relevant for a good prediction of experimental results for fuel-lean conditions and the highest DMM concentration tested (Figure 1). Additionally, results obtained with Shrestha et al.’s mechanism^7^ for C_2_H_2_ and DMM consumption are shown in Figure 3 (green lines, for interpretation of the color references, the reader is referred to the web version of the article).

As it can be seen, in the case of DMM consumption, modeling calculations for 40 bar, 70 ppm of DMM, and fuel-lean conditions (λ = 20) obtained with Shrestha et al.^7^ are in a better agreement with experimental data than those obtained with the mechanism of the present work. However, as it was previously seen in Figure 1, it fails to predict DMM consumption for 40 bar, λ = 20, and 280 ppm of DMM. This is what initially happened with our mechanism, the one previously used in the works of refs (31) and (32), and for this reason, the modifications previously described were made. Therefore, a compromise must be reached to achieve a good simulation of all the experimental conditions studied in the present work, as has been demonstrated.

**Figure 4:**
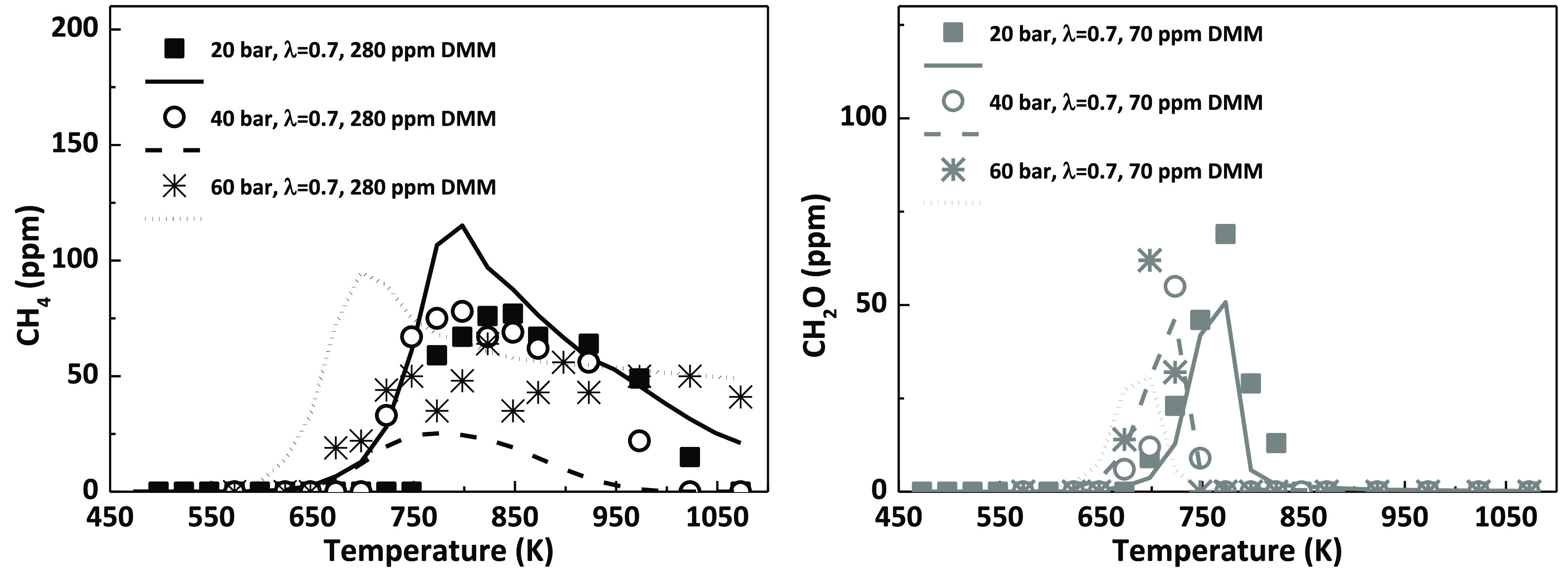
Example of the concentration profiles of other oxidation products, methane (CH_4_) and formaldehyde (CH_2_O), as a function of temperature. Conditions denoted as sets 1, 2, 7, 8, 13, and 14.

During the high-pressure oxidation of C_2_H_2_–DMM mixtures, other products have also been identified and quantified. An example of some of the results obtained is shown in Figure 4. Methane (CH_4_) has only been detected in appreciable amounts for fuel-rich conditions and the highest DMM concentration tested. A well-known issue when using gas chromatography as the main diagnostic technique is the difficulty in distinguishing between methanol (CH_3_OH) and formaldehyde (CH_2_O), as both compounds produce a very similar response. In the present work, the formation of CH_2_O is expected as has been confirmed by the match with the mechanism, as can be seen in Figure 4.

No additional species resulting from the interactions of the fuel components or through interactions of their respective reaction products have been experimentally identified.

**Figure 5:**
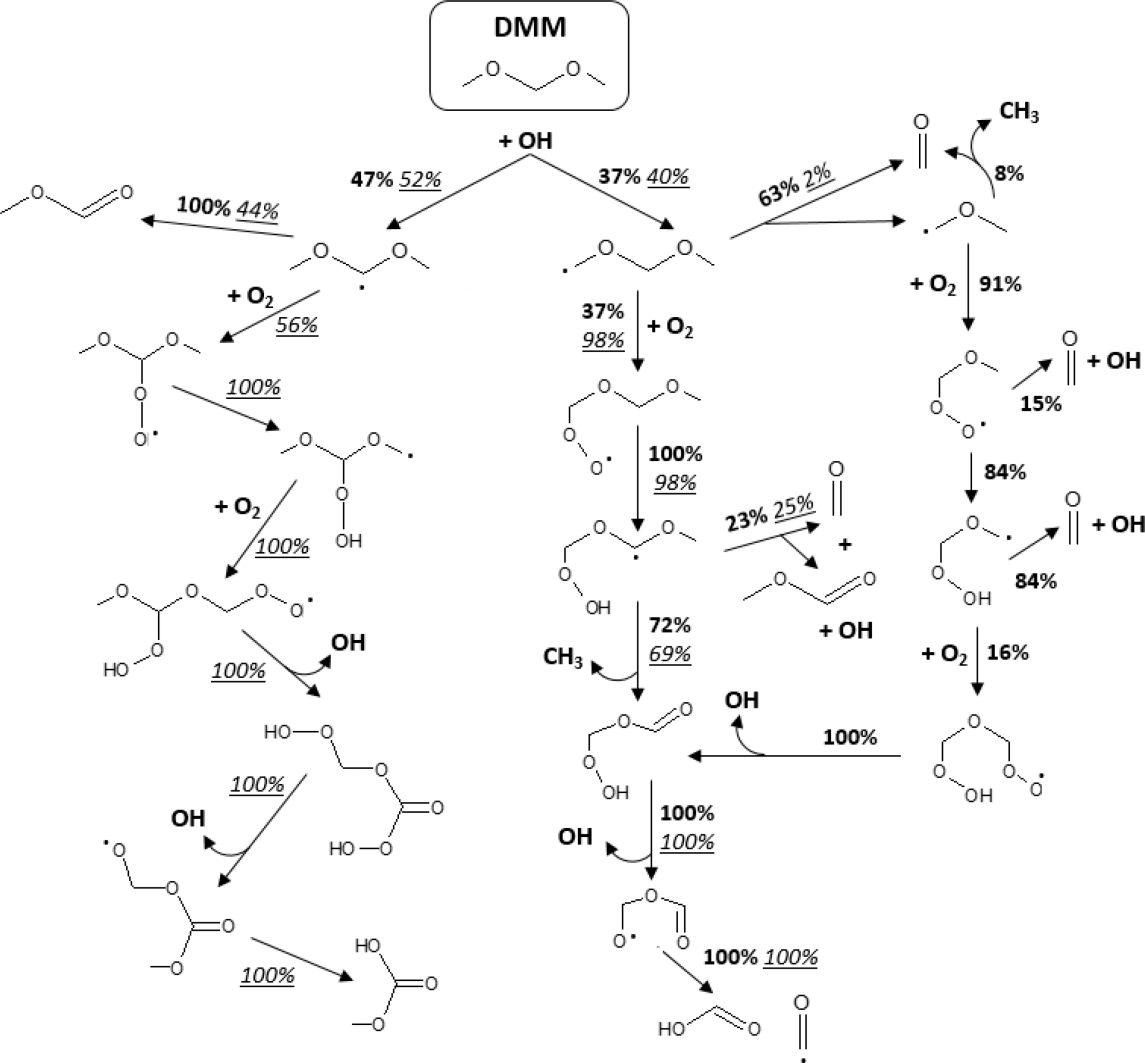
Main reaction pathways responsible of DMM consumption during the high-pressure oxidation of C_2_H_2_–DMM mixtures. Rate of productions at stoichiometric conditions (λ = 1, bold) and fuel-lean conditions (λ = 20, italics and underlined) are included. Experimental conditions: 40 bar, 70 ppm of DMM, and 698 K (λ = 1) or 648 K (λ = 20).

Once the validity of the model has been extended, both with experimental results from literature and with those corresponding to this new set of experiments, a rate of production analysis has been done for the three air excess ratios analyzed to identify the main reaction pathways. There is almost no difference between λ = 0.7 and λ = 1; therefore, in Figure 5, only percentages for stoichiometric and fuel-lean conditions are shown. The analysis has been performed for 40 bar and 70 ppm of DMM, the same conditions above shown in Figure 3. Results shown in Figure 5 correspond to the temperature and the position in the reactor that result in an approximate conversion of DMM of around 50%, i.e., 698 K for λ = 1 and 648 K for λ = 20, and a position of 1040 mm. In this work, as mentioned before, temperature profiles experimentally determined are used, so the selected position can exceed the isothermal zone. In this case, a length of 1040 mm corresponds to the end of the isothermal zone.

The consumption of DMM, for the selected conditions, proceeds through H-abstraction reactions with hydroxyl (OH) radicals as the main abstracting species over the entire temperature range studied, resulting in the formation of the two possible DMM radicals (reactions R1 and R2).
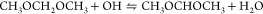
R1
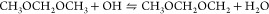
R2Under the conditions studied in this work, the formation of the dimethoxymethyl radical (CH_3_OCHOCH_3_) is slightly favored over the production of the methoxymethoxymethyl radical (CH_3_OCH_2_OCH_2_). Other radicals such as H, HO_2_, and CH_3_ participate in DMM consumption, but the contribution of these reactions is minor compared to reactions R1 and R2.

Figure 5 can be summarized as follows: there is a competition between β-scission reactions and molecular oxygen addition reactions, and the availability of oxygen in the reactant mixture tips the scales in favor of one or another type of reaction. For stoichiometric conditions, the CH_3_OCHOCH_3_ radical is completely consumed to form methyl formate and methyl radicals (reaction R3) due to the low barrier energy of the β-scission reaction that breaks the C–O bond, as stated by Jacobs et al.^24^ However, for fuel-lean conditions, there is a competition between reaction R3 and the addition of O_2_ (reaction R4). As a consequence, the formation of MF is higher for the lowest values of the air excess ratio analyzed.
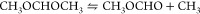
R3
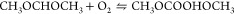
R4The dissociation energy of the C–O bond of the other DMM radical (CH_3_OCH_2_OCH_2_) (reaction R5) is comparatively higher than the energy required for reaction R3, so it is not the predominant consumption pathway of CH_3_OCH_2_OCH_2_ under stoichiometric conditions as was the case of CH_3_OCHOCH_3_ radical.
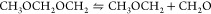
R5Homologous to the other DMM radical, this β-scission reaction (reaction R5) is in competition with O_2_ addition to form peroxyl radicals (reaction R6).
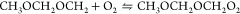
R6The reaction pathways that CH_3_OCH_2_ radicals can follow are well-known from the oxidation of DME^8,40^ and include the competition of β-scission reactions and O_2_ addition reactions, similar to those of DMM, but with a single possible site.

The main consumption routes for the peroxyl radicals (RO_2_) generated in reactions R4 and R6 include an isomerization reaction, via hydrogen atom migration forming a hydroperoxide radical (QOOH), after which a possible second O_2_ addition is possible. Only in the case of QOOH radicals formed from CH_3_OCH_2_OCH_2_ is the β-scission reaction of relative relevance compared to reaction R7.
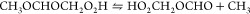
R7As represented in Figure 5, during the consumption of QOOH radicals, active hydroxyl radicals (OH) are released which participate in both DMM and C_2_H_2_ oxidation.

In the case of acetylene (C_2_H_2_), the reaction routes are the same independently of the value of λ and they have been previously described in other high-pressure oxidation works of the group.^31,32^ C_2_H_2_ consumption can be summarized in the R8–R10 reaction sequence, where OH radicals generated during the consumption of DMM play a crucial role:
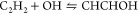
R8
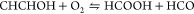
R9
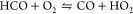
R10Since the conversion of the two fuel components, DMM and C_2_H_2_, has been adequately defined by their individual reaction subset, no further efforts have been made to identify possible cross reactions between DMM and C_2_H_2_.

**Figure 6:**
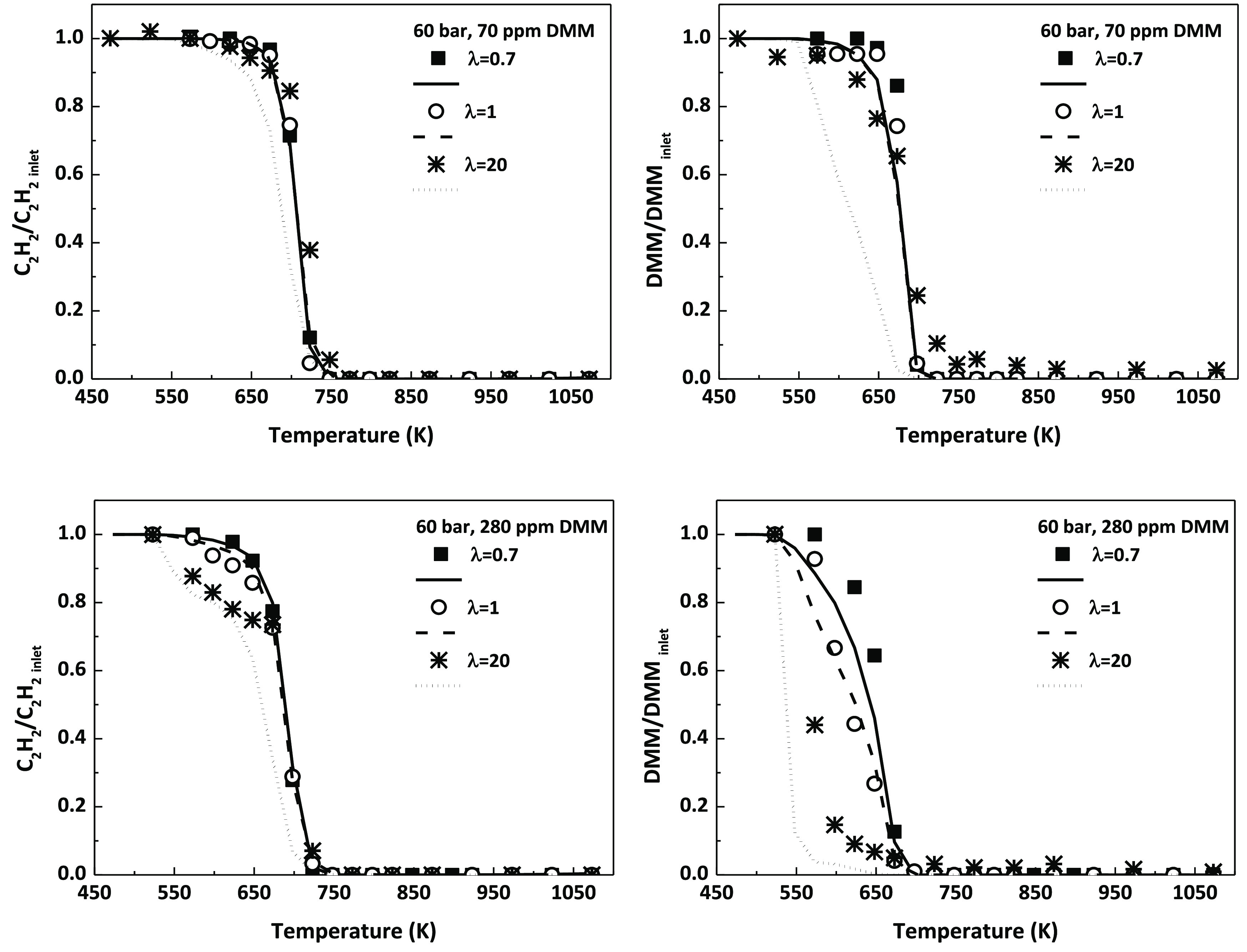
Influence of DMM inlet concentration (70 ppm, top, or 280 ppm, bottom) on the concentration profiles of C_2_H_2_ and DMM as a function of temperature for the different air excess ratios analyzed during the high-pressure C_2_H_2_–DMM mixture oxidation. Conditions denoted as sets 13–18 in Table 1.

The effect of an increase in the DMM concentration in the reactant mixture has also been evaluated. As mentioned before, two different concentrations have been tested (70 and 280 ppm, approximately) for the three values of λ established. A comparison of the results obtained for 60 bar is shown in Figure 6. Additionally, figures focusing on the effect of DMM concentration on the conversion profile of C_2_H_2_ for a given λ and 60 bar can be found in the Supporting Information (Figure S22).

An increase in the inlet DMM concentration decreases the onset temperature for C_2_H_2_ consumption. This fact also observed in the previous study of the high-pressure oxidation of C_2_H_2_–DME,^32^ where the addition of DME to the oxidation of C_2_H_2_ implies that its conversion starts at lower temperatures and, the higher the amount of DME, the lower the temperature. Both DME and DMM oxidation follow a similar pattern, including molecular oxygen addition, subsequent isomerizations and the release of OH radicals to the reactant environment which promote C_2_H_2_ conversion. The higher the amount of DMM, the higher the production of OH radicals.

A conversion of about 50% of DMM is achieved under the following conditions: λ = 20, 60 bar, 280 ppm of DMM, 548 K and a reactor position of 910 mm. In this case, the consumption of DMM proceeds through H-abstraction reactions (reactions R1 and R2) as mentioned before. Once both DMM radicals are formed, there is no competition between β-scission and O_2_ addition reactions; the addition of molecular oxygen is clearly favored. The DMM reaction pathways, identified and proposed in the previous DMM oxidation study in JSR of Vermeire et al.,^23^ indicated that CH_3_OCHOCH_3_ radical, whose formation is favored over the production of CH_3_OCH_2_OCH_2_, is completely consumed by a β-scission reaction because of the low energy barrier of this reaction, which makes it so fast that it is not possible a competition. However, this is true under stoichiometric conditions, because an increase in the concentration of O_2_ or the DMM radical will make the O_2_ addition reaction faster enough to be the most favored reaction.

**Figure 7:**
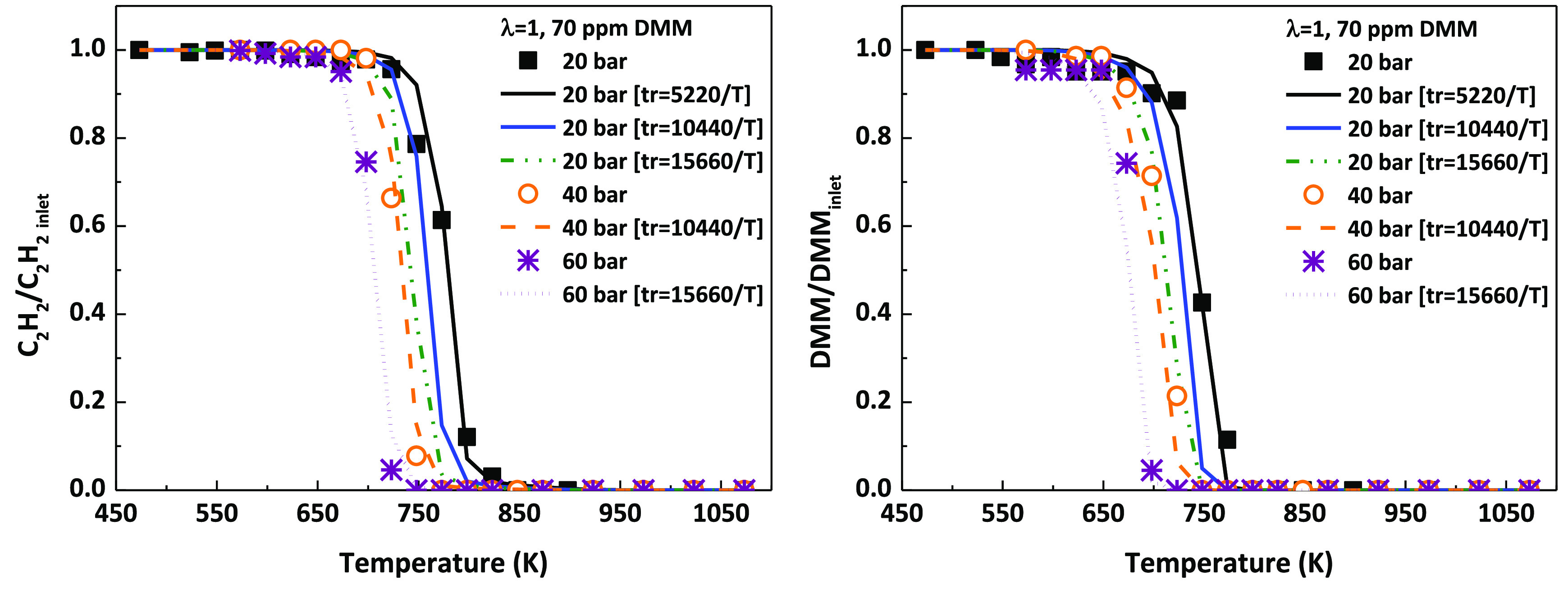
Influence of pressure and gas residence time (*t*_r_) on C_2_H_2_–DMM mixture oxidation (70 ppm of DMM) under stoichiometric conditions (λ = 1).

In this work, oxidation experiments have been performed in a wide range of high-pressure conditions (20, 40, and 60 bar). Figure 7 shows the results at different pressures on the C_2_H_2_ and DMM conversion for stoichiometric conditions and 70 ppm of DMM. As it can be seen, the onset temperature for both C_2_H_2_ and DMM conversion is shifted to lower temperatures as the working pressure is increased. We are aware of the fact that when pressure is increased, for the same temperature, the gas residence time also increases according to eq 1. In order to try to elucidate which of the effects is predominant, modeling calculations have been performed while maintaining the pressure and increasing the gas residence time. Results of this evaluation are also included in Figure 7 (blue and green lines, for interpretation of the color references, the reader is referred to the web version of the article).

Results indicate that both the pressure and the gas residence time have an effect on C_2_H_2_ and DMM conversion, which are shifted to lower temperatures if any of these variables increased while keeping the other one constant. Similar to what has been observed in other C_2_H_2_–oxygenate mixture oxidation studies, such as C_2_H_2_–DME.^32^ As a consequence, the change in the onset temperature for the C_2_H_2_ and DMM conversion can be attributed both to the increase in pressure, and the consequent increase in the concentration of reactants, and to the related increase in the gas residence time.

**Figure 8:**
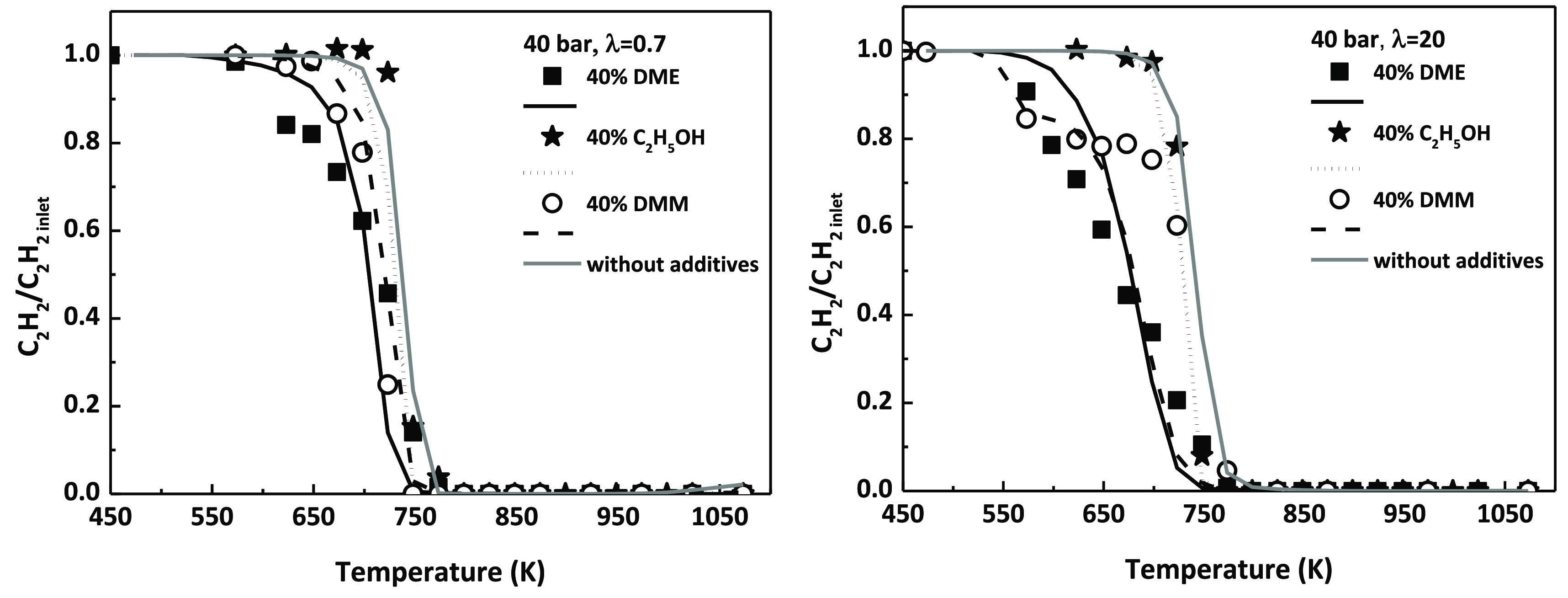
Effect of the addition of different additives (DME, ethanol, and DMM) on the high-pressure (40 bar) oxidation of C_2_H_2_, for λ = 0.7 (left) and λ = 20 (right).

Finally, the effect of the addition of different oxygenates on the high-pressure oxidation of C_2_H_2_ has been evaluated. Therefore, results obtained during the high-pressure oxidation of C_2_H_2_–ethanol/DME/DMM mixtures, as prospective additives, in the same experimental setup,^31,32^ will be compared. Figure 8 shows a comparison for two different values of the air excess ratio (λ), fuel-rich and fuel-lean conditions, and 40 bar (value of pressure experimentally analyzed for all the compounds under the same conditions). For the C_2_H_2_ high-pressure oxidation in the absence of additives, modeling calculations with the present mechanism have been performed and included in Figure 8.

The addition of ethanol has almost no effect on the oxidation of C_2_H_2_, the predicted C_2_H_2_ concentration profile remains almost the same as without any additive, while the presence of an ether, DME or DMM, shifts the conversion of C_2_H_2_ to lower temperatures. The chemical structure, and the favorable formation of QOOH radicals, clearly influences the reactivity at low temperatures (550–750 K) as stated by Yang et al.^41^ in a recent review on the interaction of oxygenates on hydrocarbon combustion when comparing studies of the isomers DME and ethanol.

The shifting in the onset temperature for C_2_H_2_ conversion is more significant for DME addition, the simplest ether considered, and it is more noticeable for fuel-lean conditions. Moreover, the oxidation of C_2_H_2_ toward CO and CO_2_ is favored by the addition of oxygenated compounds, instead of following reaction pathways which may lead to the formation of soot, due to an increase in the O/OH radical pool composition because of the oxygen present in such compounds.

## 4 Conclusions

In this work, high-pressure (20, 40, and 60 bar) oxidation experiments of acetylene (C_2_H_2_) and dimethoxymethane (DMM) mixtures have been performed in a tubular flow reactor. In addition to pressure, several air excess ratios, λ, from fuel-rich to fuel-lean conditions, have been evaluated along with two different concentrations of DMM, 70 and 280 ppm, for a constant concentration of 700 ppm of C_2_H_2_. This highly valuable experimental data set, which extends the existing database, has been used to validate and update our chemical kinetic mechanism with recent theoretical calculations on DMM pyrolysis and oxidation.

Under fuel-lean conditions (λ = 20), the presence of DMM in the reactant mixture promotes C_2_H_2_ oxidation, shifting its conversion to lower temperatures compared to fuel-rich and stoichiometric conditions. This fact is more evident for the higher concentration of DMM tested, 280 ppm. In general, the model successfully reproduces the trends experimentally observed, although there are some discrepancies between experimental results and modeling calculations for fuel-lean conditions and the lowest concentration of DMM tested (70 ppm).

The analysis of the main consumption routes (rate of production analysis) helps to explain the evidence observed. In the case of DMM, it is consumed by H-abstraction reactions with OH radicals to form CH_3_OCHOCH_3_ and CH_3_OCH_2_OCH_2_ radicals, with the formation of the first one slightly favored. Once both radicals have been produced, β-scission and O_2_-addition reactions compete. This competition highly depends on the oxygen availability; i.e., for fuel-rich and stoichiometric conditions, β-scission reactions are favored, whereas for fuel-lean conditions O_2_-addition routes predominate which include subsequent isomerizations and OH radicals release which promote C_2_H_2_ oxidation.

This work can be included within a more extensive project on the influence of the addition of different oxygenates (ethanol and two ethers, DME and DMM), as prospective additives, on the high-pressure oxidation of C_2_H_2_. Results indicate that the presence of any of the ethers, DME or DMM, promotes C_2_H_2_ oxidation, shifting its conversion to lower temperatures. However, the addition of ethanol produces almost no effect on the conversion of C_2_H_2_ and its predicted concentration profile remains as without any additive.

## Supporting Information Available

Chemical kinetic mechanism (TXT)
Thermodynamic data (TXT)
Experimental data (XLSX)
Temperature profiles, gas chromatography spectra, model performance for literature experimental data sets and effect of an increase in the DMM concentration (PDF)
