# Supplementary material for: Experimental and Modeling Evaluation of Dimethoxymethane as an Additive for High-Pressure Acetylene Oxidation
Source: J Phys Chem A. 2022 Sep 1;126(36):6253–63. doi: 10.1021/acs.jpca.2c03130 (PMC9483988; doi:10.1021/acs.jpca.2c03130)
Supplement: Supplementary file 4 — jp2c03130_si_004.pdf [file jp2c03130_si_004.pdf]

SUPPORTING INFORMATION

Experimental and Modeling Evaluation of Dimethoxymethane as an Additive for High-Pressure Acetylene Oxidation

Lorena Marrodán, Ángela Millera, Rafael Bilbao, María U. Alzueta*

Aragón Institute of Engineering Research (I3A). Department of Chemical and Environmental Engineering. University of Zaragoza. R+D building. Río Ebro Campus. 50018 Zaragoza. Spain
*uxue@unizar.es

1. Temperature profiles inside the reactor S1
2. Gas chromatography spectra S3
3. Model performance for experimental data sets found in literature S5
   a. Dimethoxymethane oxidation in a jet-stirred reactor (JSR) S5
   b. Dimethoxymethane oxidation in an atmospheric-pressure tubular-flow reactor S9
   c. Dimethoxymethane oxidation in a high-pressure tubular-flow reactor S14
   d. Ignition delay times of DMM S17
   e. Acetylene oxidation in a high-pressure tubular-flow reactor S18
4. Effect of an increase in the DMM concentration in the reactant mixture on C_2H_2 conversion S19
5. References S19

1. Temperature profiles inside the reactor

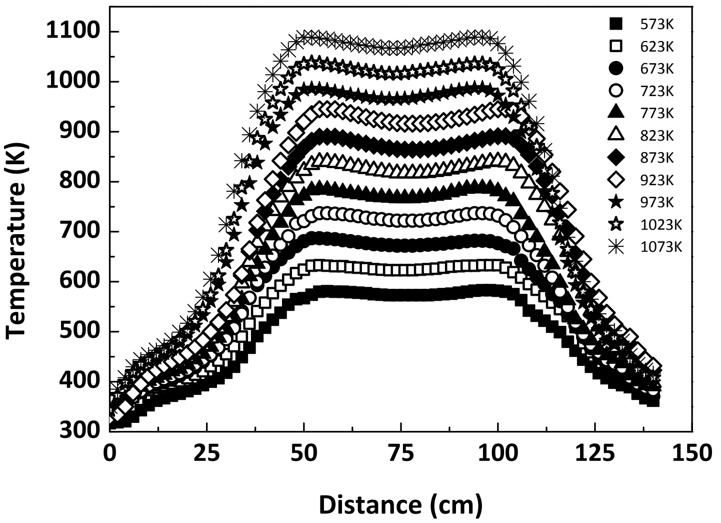

Figure S1. Temperature profiles for different nominal temperatures as a function of distance for a flow rate of 1 L (STP)/min and 20 bar.

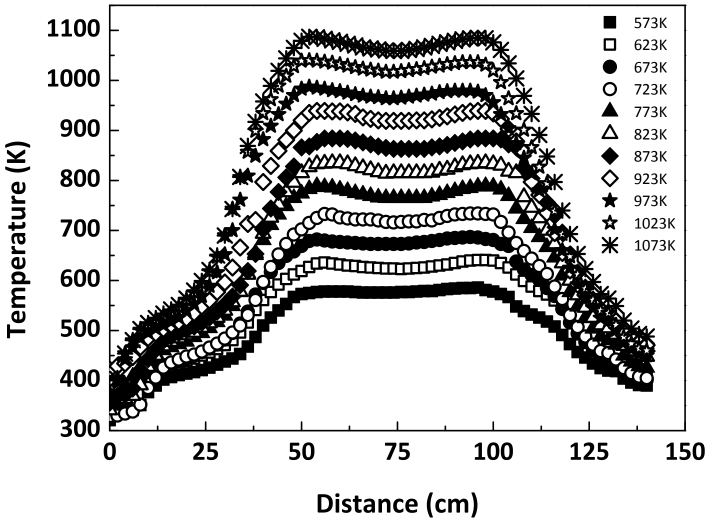

Figure S2. Temperature profiles for different nominal temperatures as a function of distance for a flow rate of 1 L (STP)/min and 40 bar.

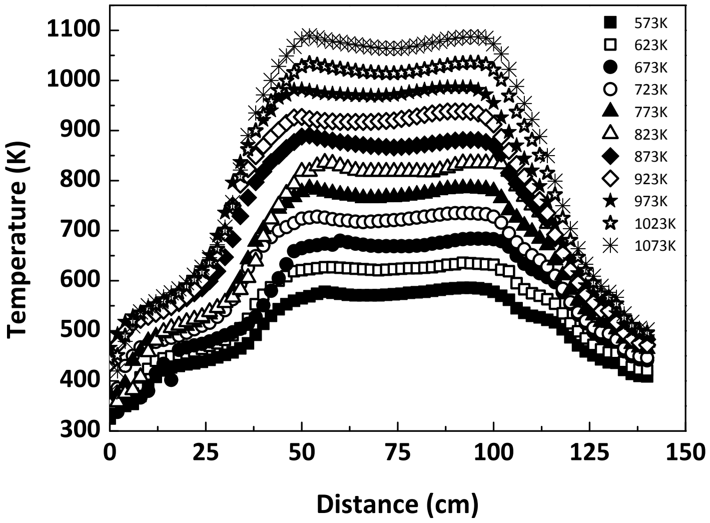

Figure S3. Temperature profiles for different nominal temperatures as a function of distance for a flow rate of 1 L (STP)/min and 60 bar.

2. Gas chromatography spectra

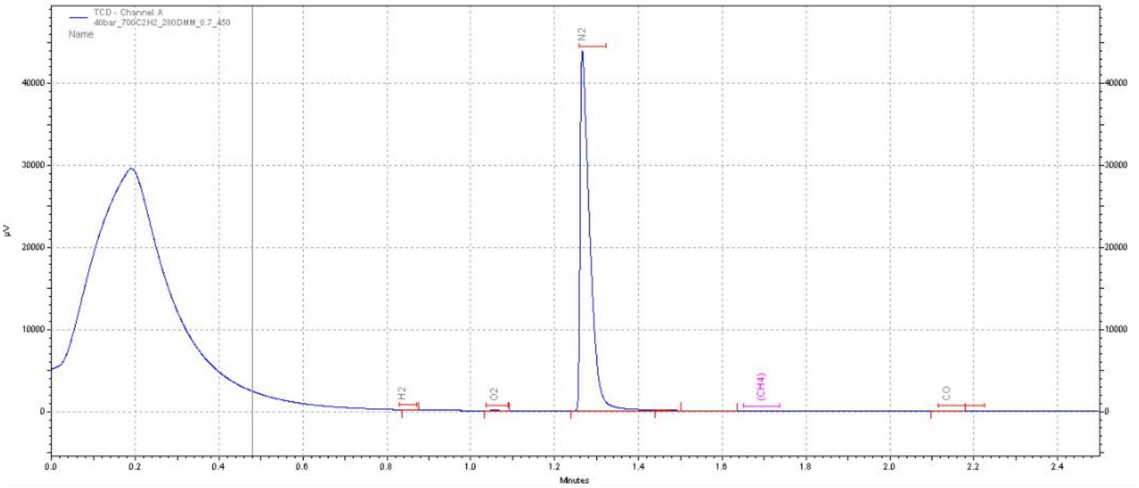

Figure S4. Example of chromatogram obtained during the high-pressure oxidation of C_2H_2-DMM mixtures for module A (molecular sieve column with a Plot U precolumn).

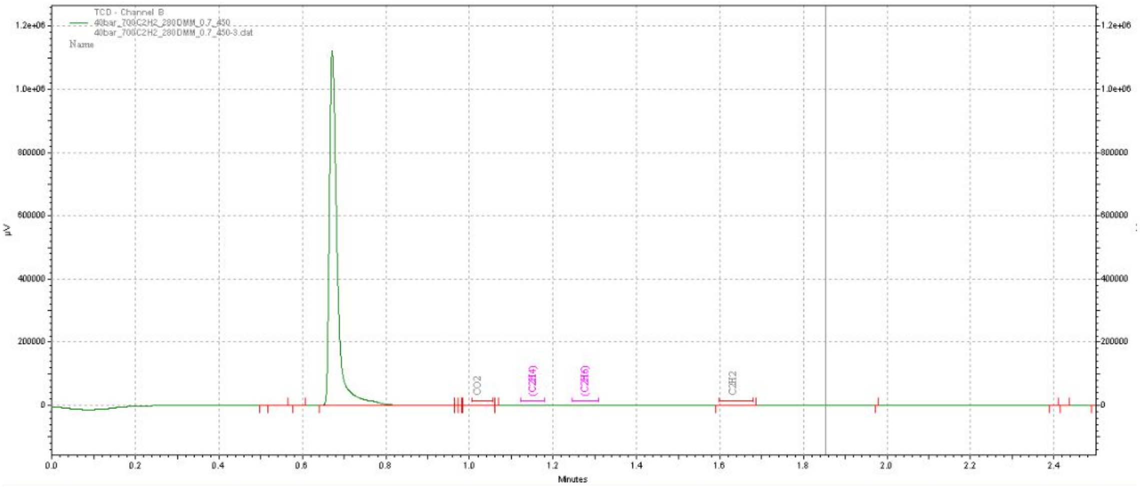

Figure S5. Example of chromatogram obtained during the high-pressure oxidation of C_2H_2-DMM mixtures for module B (PPU column with a Plot Q precolumn).

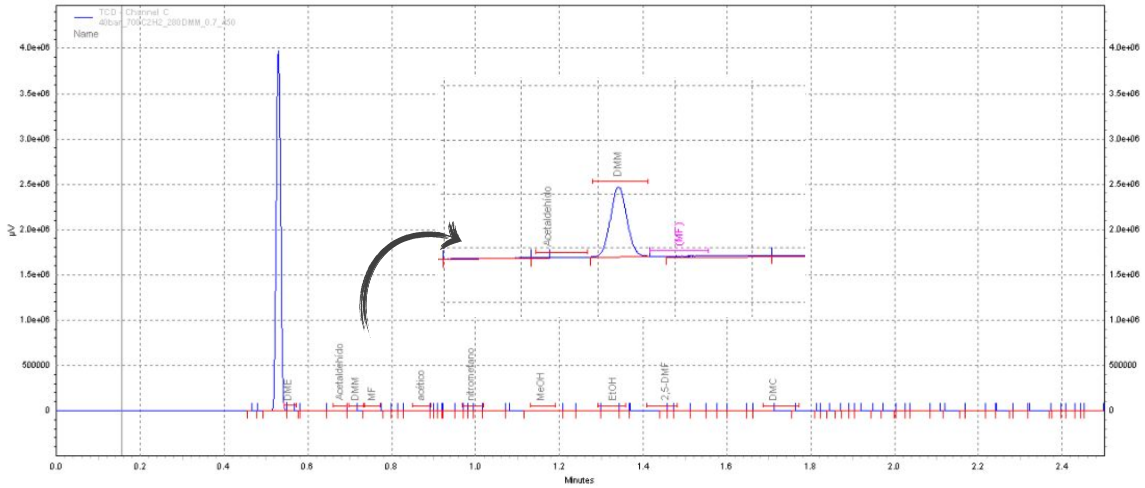

Figure S6. Example of chromatogram obtained during the high-pressure oxidation of C_2H_2-DMM mixtures for module C (Stabilwax DB column).

3. Model performance for experimental data sets found in literature

a. Dimethoxymethane oxidation in a jet-stirred reactor (JSR)

Experiments reported by Vermeire et al.[23] performed in a quartz jet-stirred reactor have also been used to validate the kinetic mechanism. Four different equivalence ratios have been investigated, ø=∞ (pyrolysis), ø=2, ø=1 and ø=0.25. Simulations have been performed with the continuous stirred-tank reactor of the Chemkin-Pro software package[38]. Results of the comparison of model calculations and experimental data are shown in Figures S7-S10.

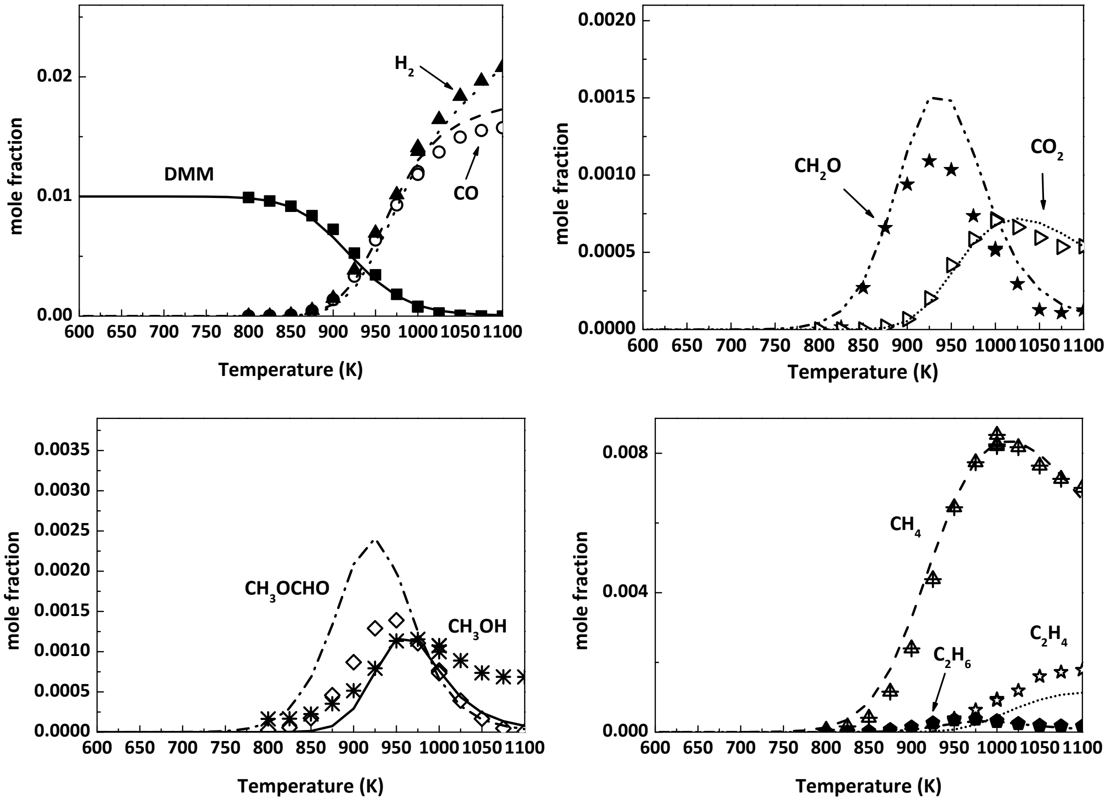

Figure S7. Species mole fractions as a function of temperature for the pyrolysis of DMM. Experimental results (symbols) reported by Vermeire et al.[23], and modeling calculations (lines) obtained with the present mechanism are compared. Experimental conditions: 1.07 bar, residence time=2.83 s and DMM inlet mole fraction=0.01.

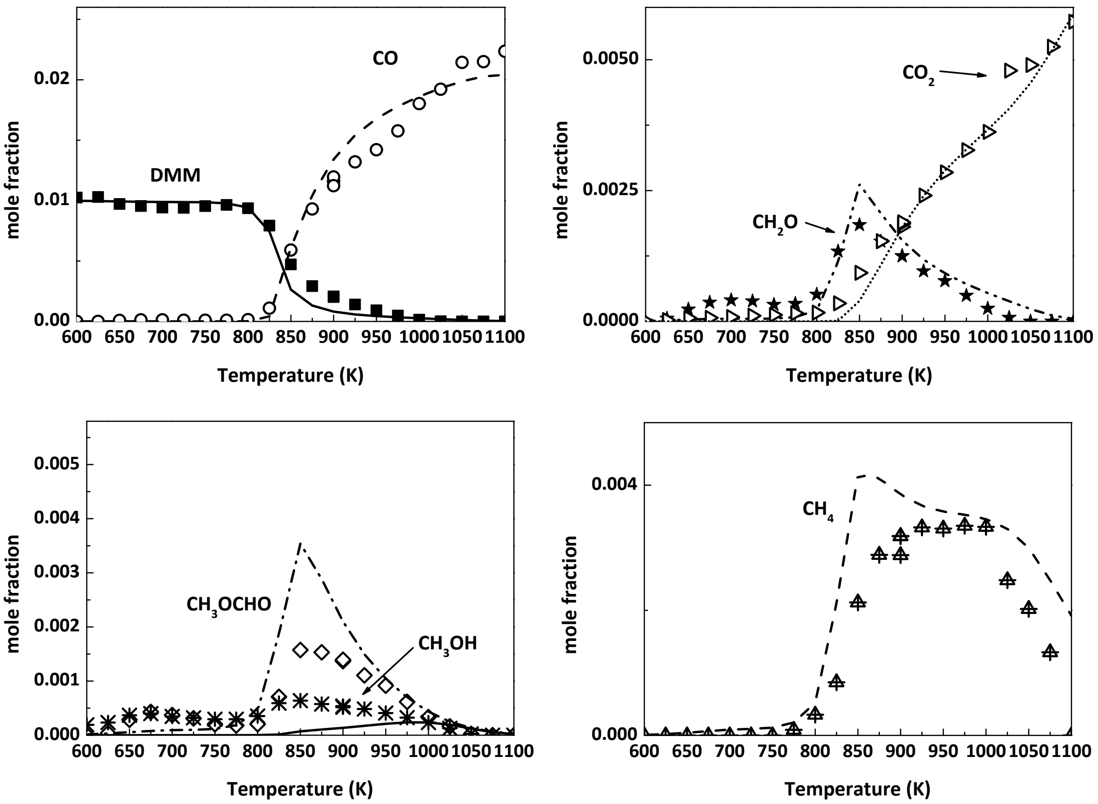

Figure S8. Species mole fractions as a function of temperature for the oxidation of DMM. Experimental results (symbols) reported by Vermeire et al.[23], and modeling calculations (lines) obtained with the present mechanism are compared. Experimental conditions: ø=2, 1.07 bar, residence time=2.83 s and DMM inlet mole fraction=0.01.

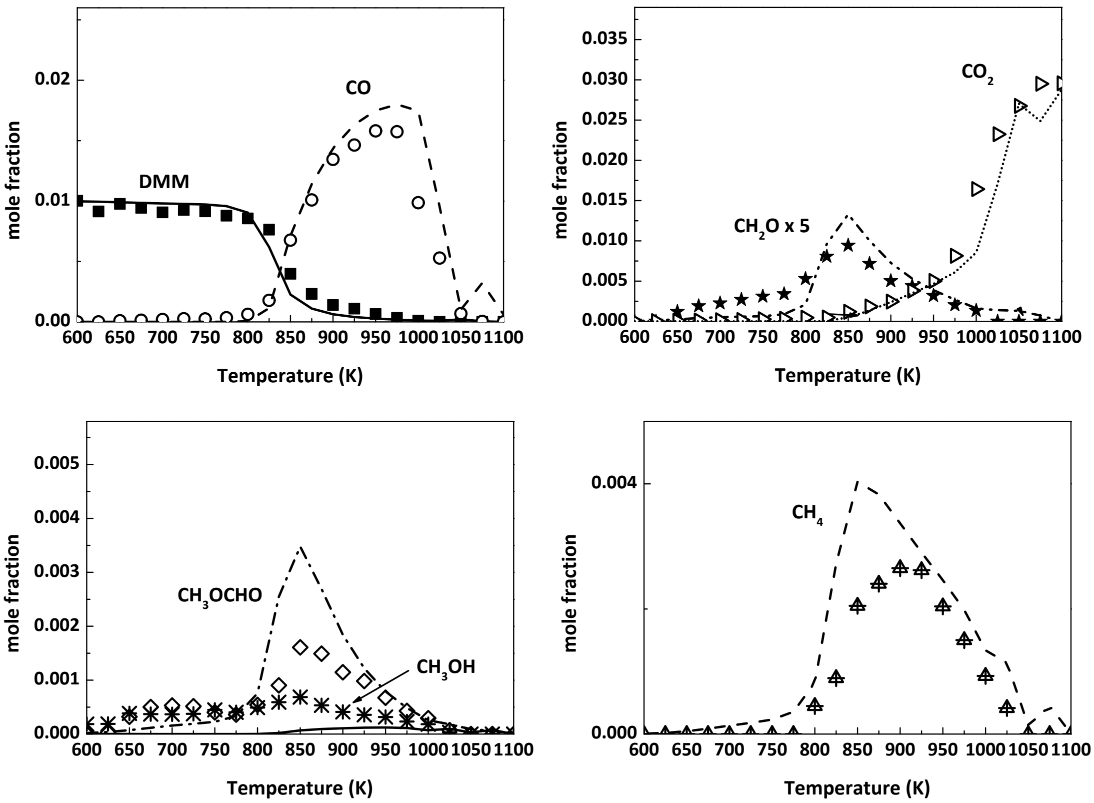

Figure S9. Species mole fractions as a function of temperature for the oxidation of DMM. Experimental results (symbols) reported by Vermeire et al.[23], and modeling calculations (lines) obtained with the present mechanism are compared. Experimental conditions: ϕ=1, 1.07 bar, residence time=2.83 s and DMM inlet mole fraction=0.01.

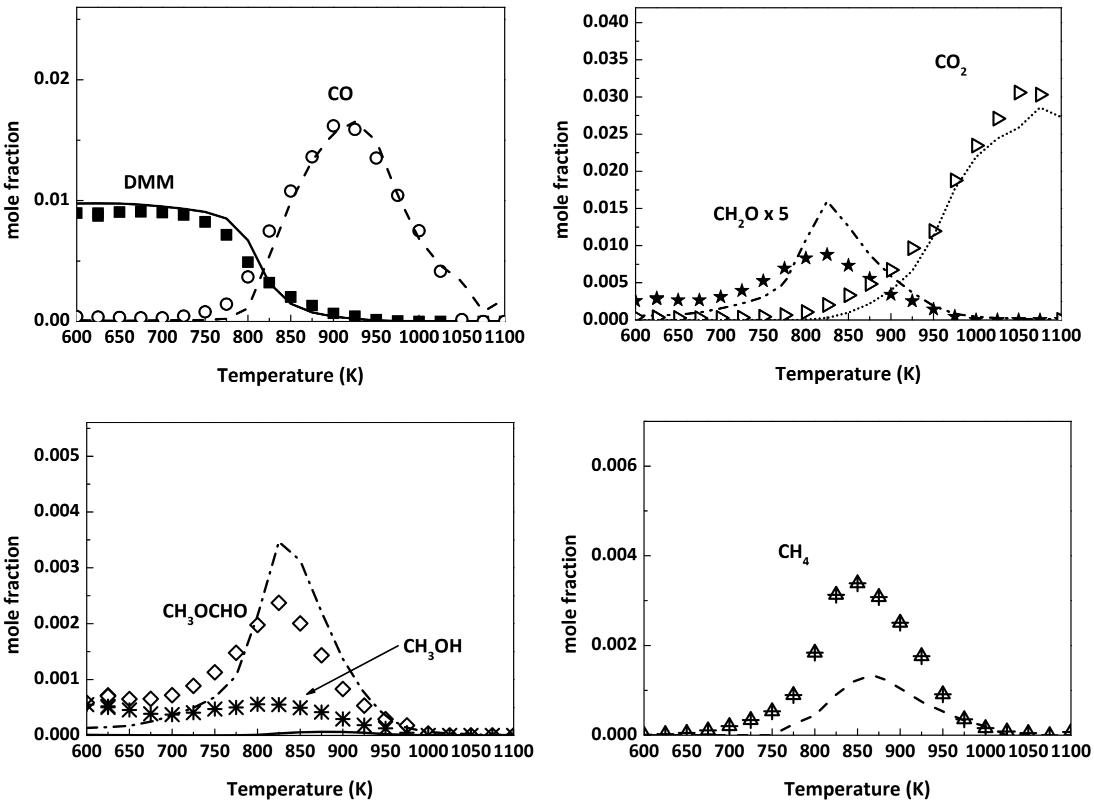

Figure S10. Species mole fractions as a function of temperature for the oxidation of DMM. Experimental results (symbols) reported by Vermeire et al.[23], and modeling calculations (lines) obtained with the present mechanism are compared. Experimental conditions: ϕ=0.25, 1.07 bar, residence time=2.83 s and DMM inlet mole fraction=0.01.

b. Dimethoxymethane oxidation in an atmospheric-pressure tubular-flow reactor

The kinetic mechanism has been validated with experiments reported by Marrodán et al.[21] performed in a tubular-flow reactor at atmospheric pressure from pyrolysis to fuel-lean conditions, i.e. the air excess ratio was varied from λ=0 to λ=35. Simulations have been performed with the plug-flow reactor module of the Chemkin-Pro software package[38]. Results of the comparison of model calculations and experimental data are shown in Figures S11-S15.

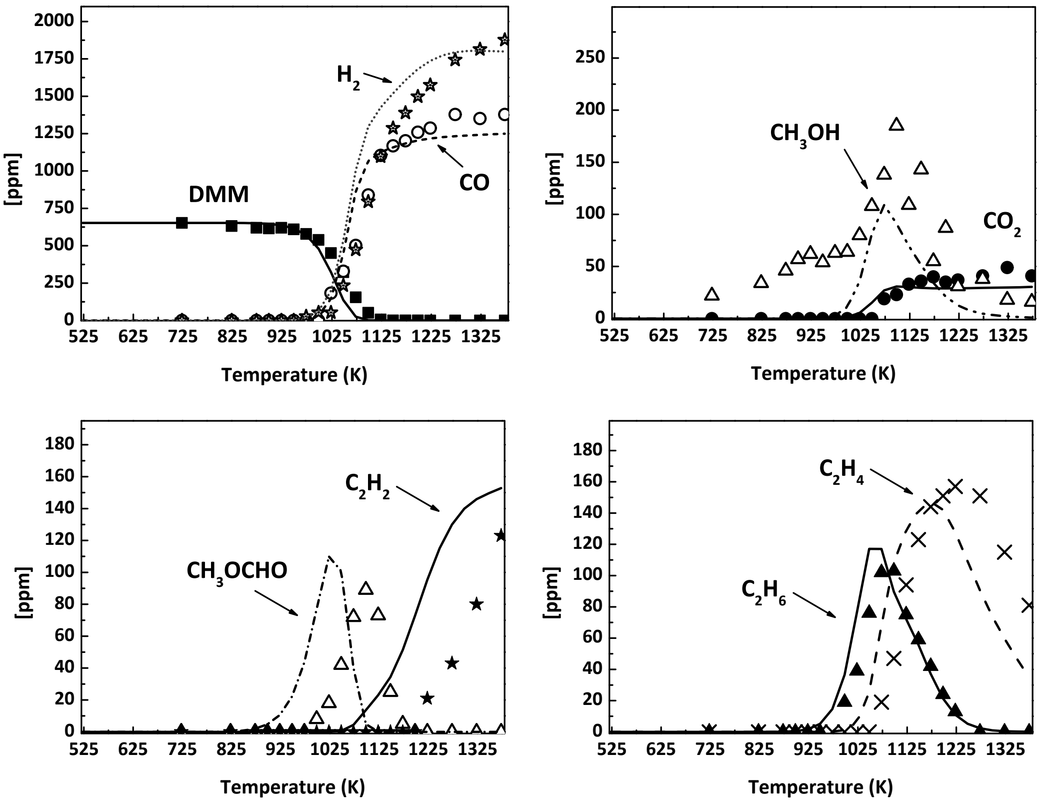

Figure S11. Species concentration as a function of temperature for the pyrolysis of DMM. Experimental results (symbols) reported by Marrodán et al.[21], and modeling calculations (lines) obtained with the present mechanism are compared for atmospheric pressure.

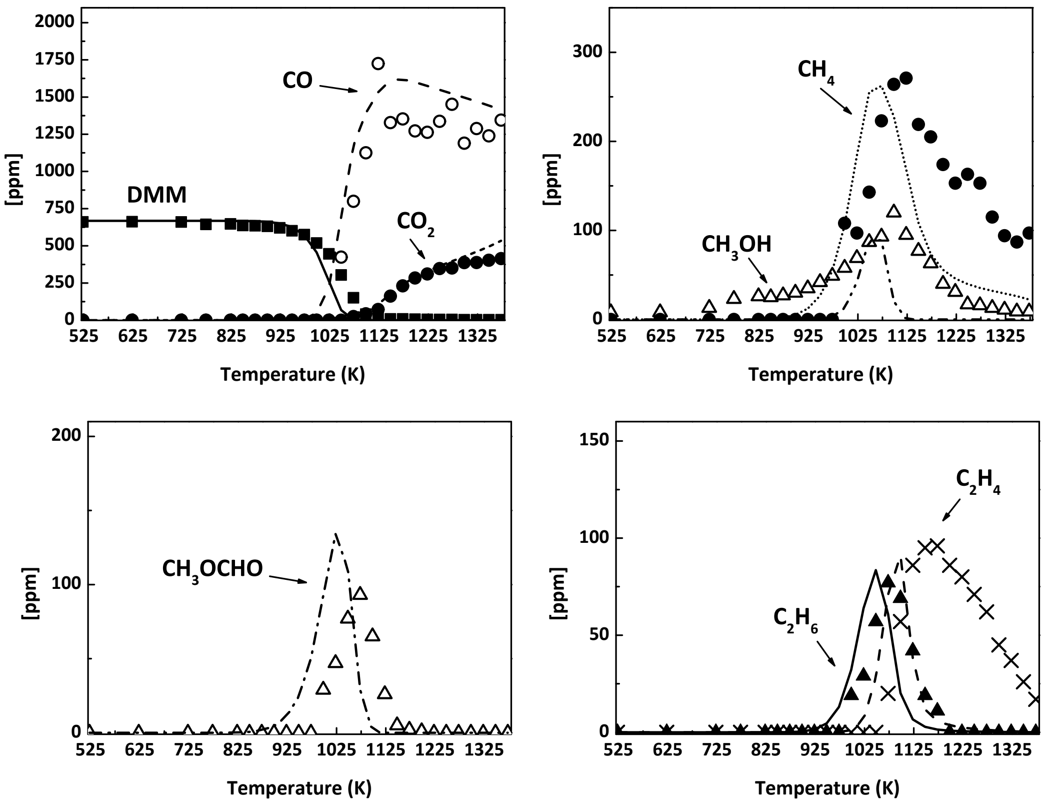

Figure S12. Species concentration as a function of temperature for the oxidation of DMM, λ=0.4. Experimental results (symbols) reported by Marrodán et al.[21], and modeling calculations (lines) obtained with the present mechanism are compared for atmospheric pressure.

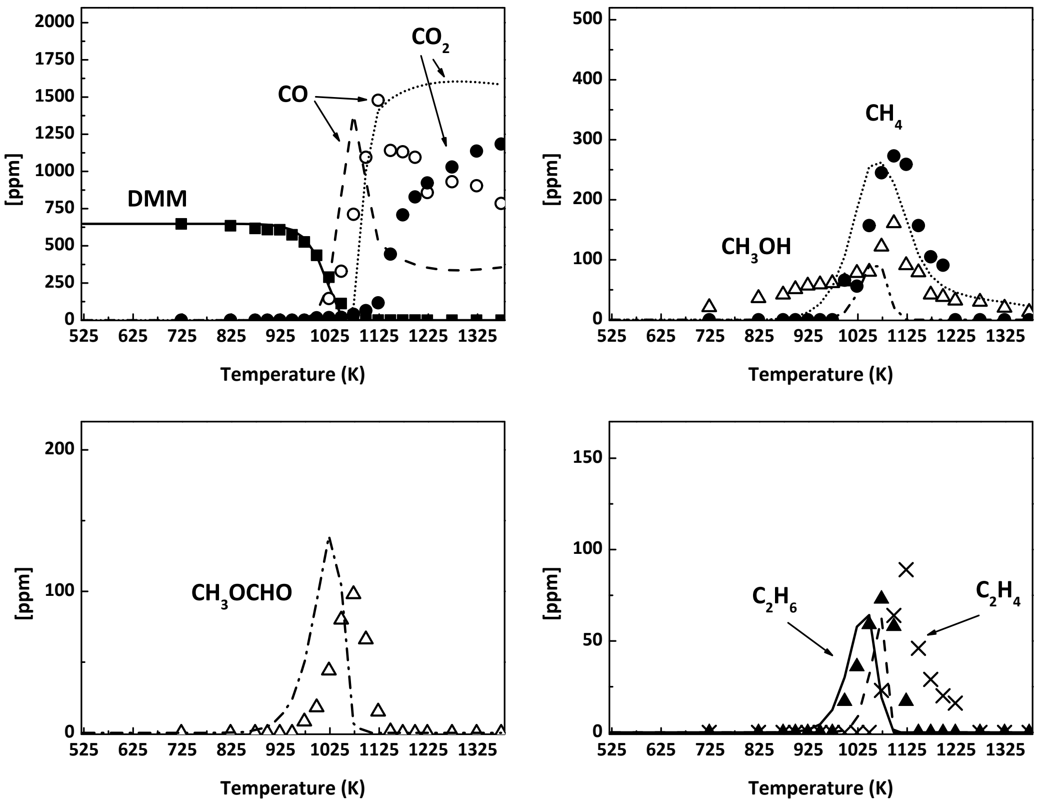

Figure S13. Species concentration as a function of temperature for the oxidation of DMM, λ=0.7. Experimental results (symbols) reported by Marrodán et al.[21], and modeling calculations (lines) obtained with the present mechanism are compared for atmospheric pressure.

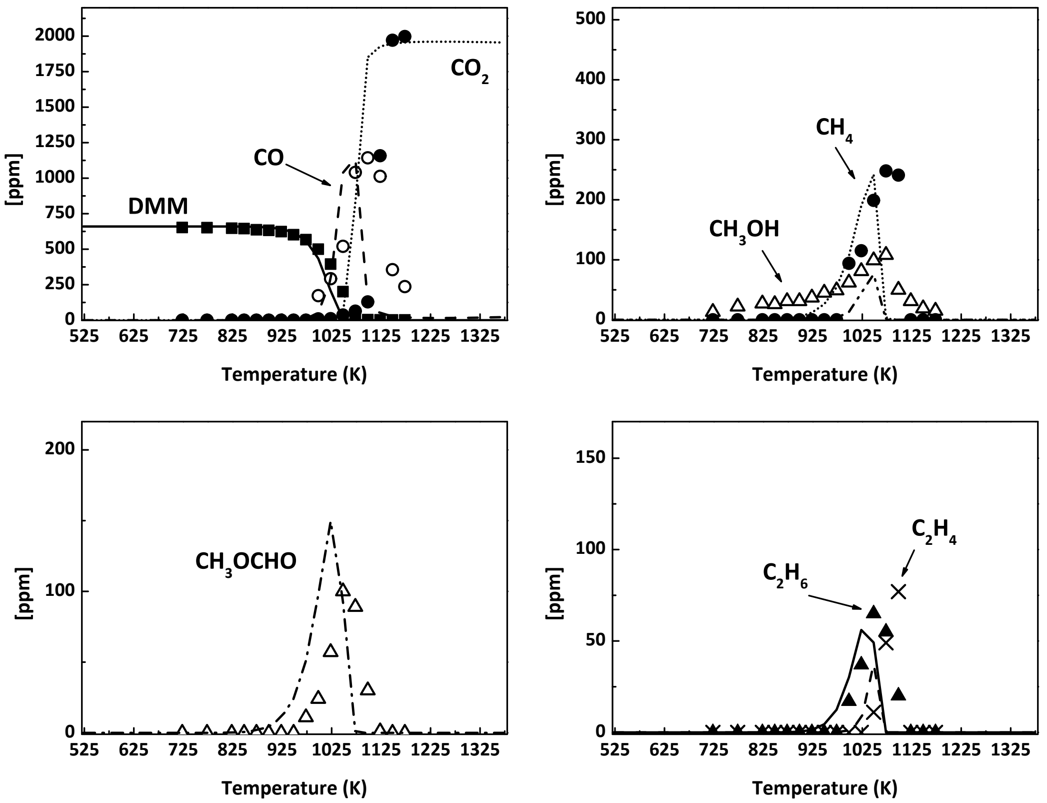

Figure S14. Species concentration as a function of temperature for the oxidation of DMM, λ=1. Experimental results (symbols) reported by Marrodán et al.[21], and modeling calculations (lines) obtained with the present mechanism are compared for atmospheric pressure.

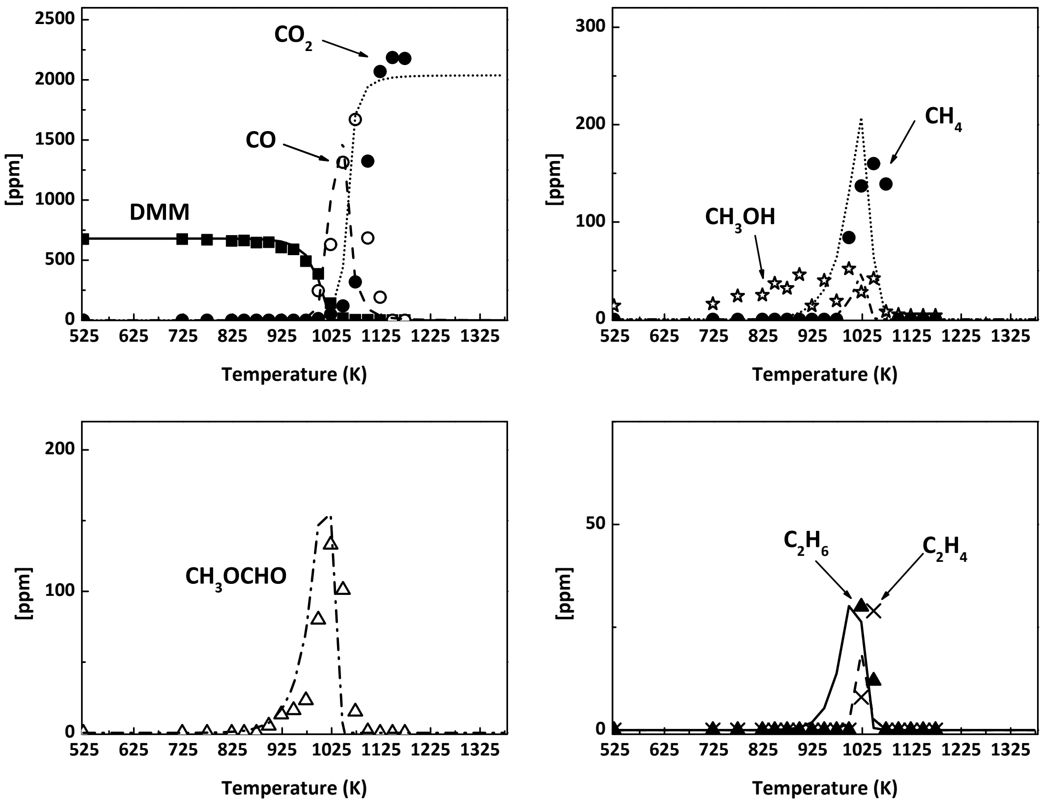

Figure S15. Species concentration as a function of temperature for the oxidation of DMM, λ=35. Experimental results (symbols) reported by Marrodán et al.[21], and modeling calculations (lines) obtained with the present mechanism are compared for atmospheric pressure.

c. Dimethoxymethane oxidation in a high-pressure tubular-flow reactor

The kinetic mechanism has also been validated with experiments reported by Marrodán et al.[20] performed in a tubular-flow reactor at high pressure (20-60 bar). The air excess ratio was varied from λ=0.7 to λ=20. Simulations have been performed with the plug-flow reactor module of the Chemkin-Pro software package[38]. Results of the comparison of model calculations and experimental data are shown in Figures S16-S18.

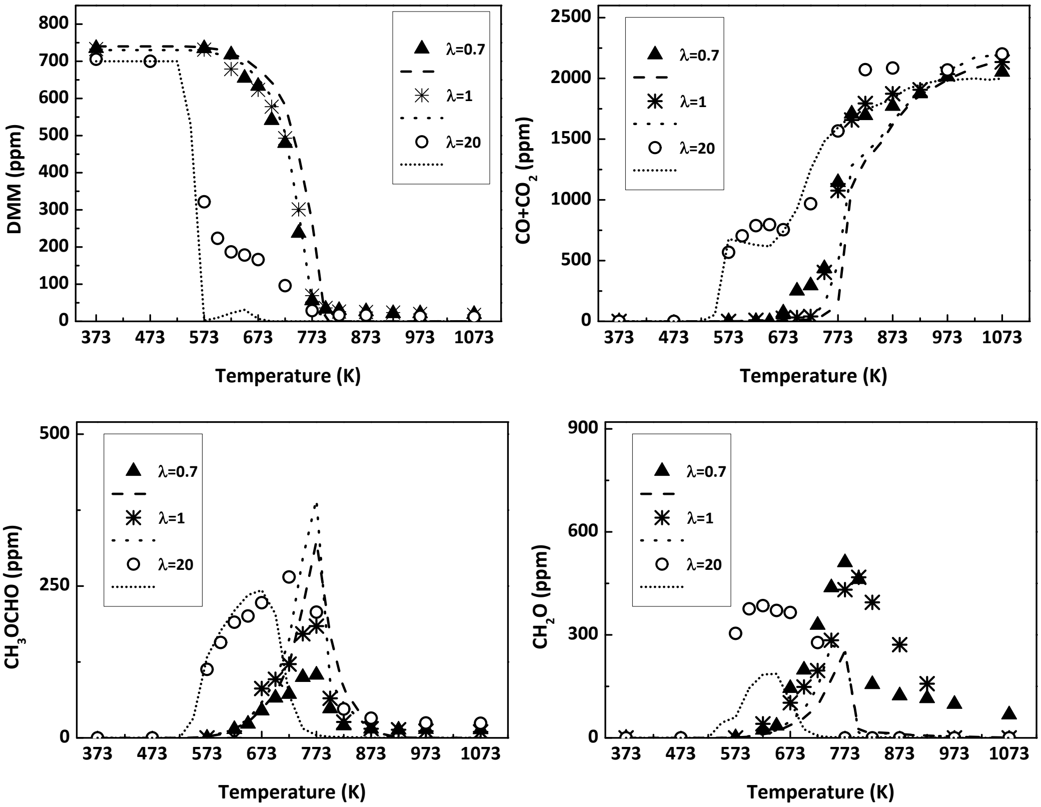

Figure S16. Species concentration as a function of temperature for the oxidation of DMM at 20 bar. Experimental results (symbols) reported by Marrodán et al.[20], and modeling calculations (lines) obtained with the present mechanism are compared for different air excess ratios (λ=0.7, λ=1 and λ=20).

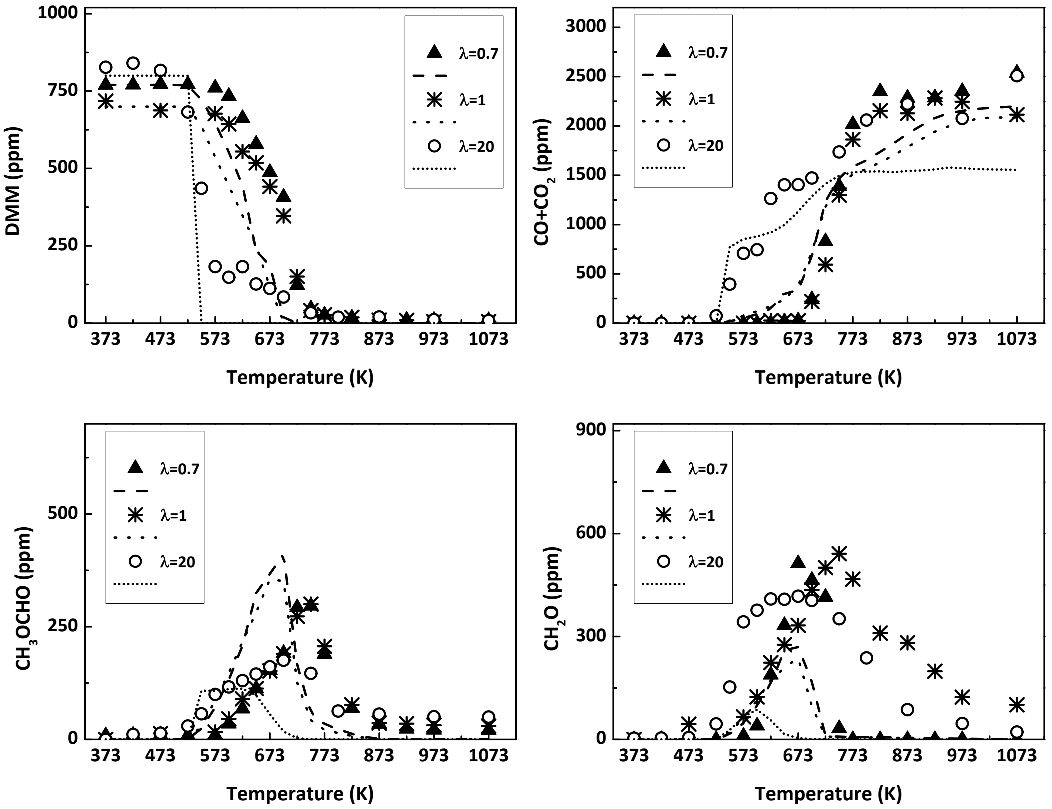

Figure S17. Species concentration as a function of temperature for the oxidation of DMM at 40 bar. Experimental results (symbols) reported by Marrodán et al.[20], and modeling calculations (lines) obtained with the present mechanism are compared for different air excess ratios (λ=0.7, λ=1 and λ=20).

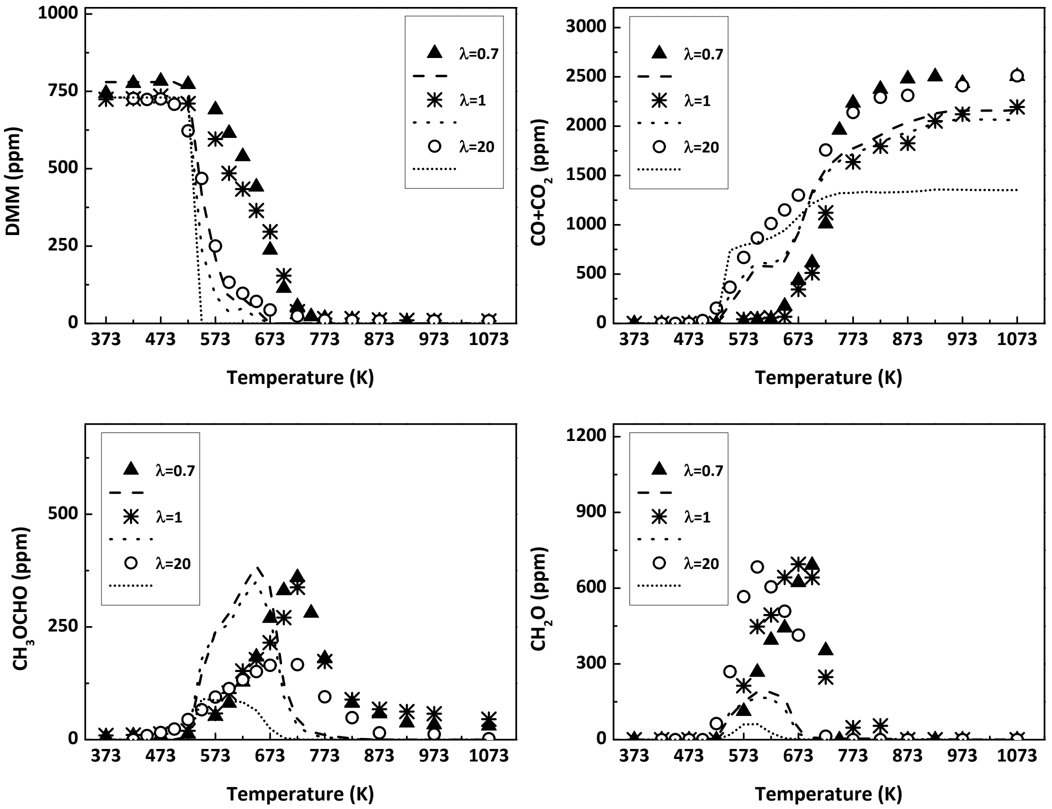

Figure S18. Species concentration as a function of temperature for the oxidation of DMM at 60 bar. Experimental results (symbols) reported by Marrodán et al.[20], and modeling calculations (lines) obtained with the present mechanism are compared for different air excess ratios (λ=0.7, λ=1 and λ=20).

d. Ignition delay times of DMM

Ignition delay times reported by Li et al.[26] measured in a shock tube at pressures 1 and 4 atm, for equivalence ratios of 0.5, 1 and 2 have also been used to validate the kinetic mechanism. Simulations have been performed with the closed homogeneous reactor of the Chemkin-Pro software package[38]. Results of the comparison of model calculations and experimental data are shown in Figures S19 and S20.

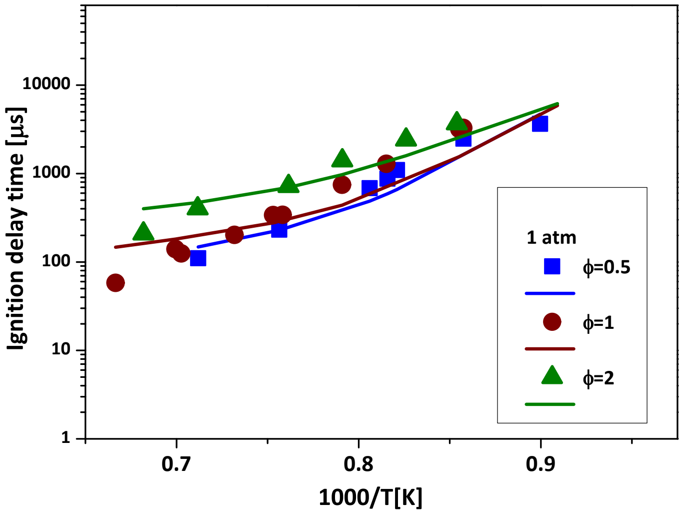

Figure S19. Comparison of experimental and predicted ignition delay times of DMM. Experimental results (symbols) reported by Li et al.[26], and modeling calculations (lines) obtained with the present mechanism are compared for different equivalence ratios (ø=0.5, ø=1 and ø=2) and 1 atm.

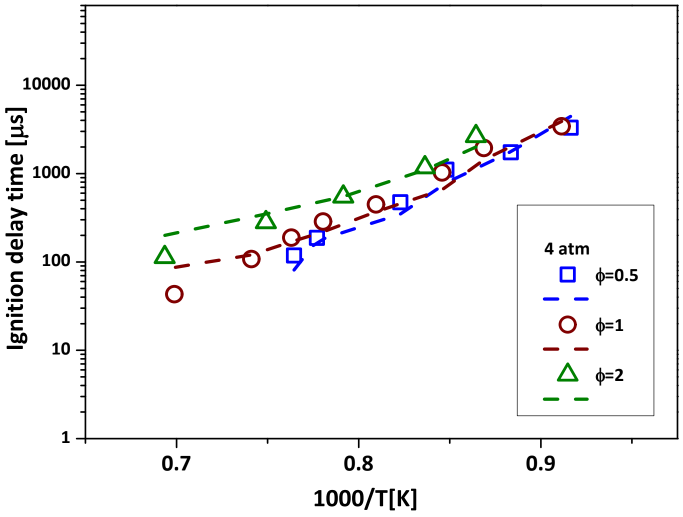

Figure S20. Comparison of experimental and predicted ignition delay times of DMM. Experimental results (symbols) reported by Li et al.[26], and modeling calculations (lines) obtained with the present mechanism are compared for different equivalence ratios (ø=0.5, ø=1 and ø=2) and 4 atm.

e. Acetylene oxidation in a high-pressure tubular-flow reactor

Experiments reported by Giménez et al.[39], performed in a tubular-flow reactor, have also been used to validate the kinetic mechanism. Two different air excess ratios (λ=0.99 and λ=19.4) for two pressures, 59.6 and 49.6 bar, respectively, have been tested. Simulations have been performed with the closed homogeneous reactor of the Chemkin-Pro software package[38] by fixing the gas residence time inside the reactor. Results of the comparison of model calculations and experimental data are shown in Figure S21.

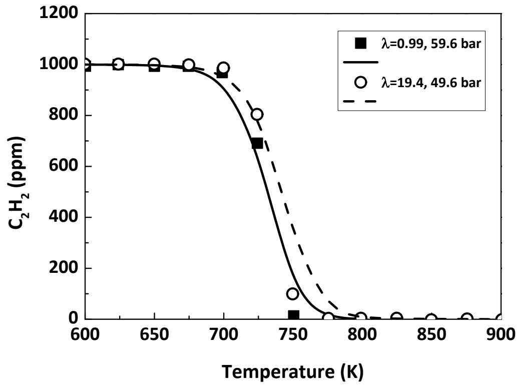

Figure S21. Acetylene concentration as a function of temperature during its oxidation at high-pressure (59.6 and 49.6 bar). Experimental results (symbols) reported by Giménez et al.[39], and modeling calculations (lines) obtained with the present mechanism are compared for different air excess ratios (λ=0.99 and λ=19.4).

4. Effect of an increase in the DMM concentration in the reactant mixture on C_2H_2 conversion

The effect of an increase in the DMM concentration in the reactant mixture has also been evaluated. Two different concentrations have been tested (70 and 280, approximately) for the three values of λ established. A comparison of the results obtained for 60 bar is shown in Figure S22.

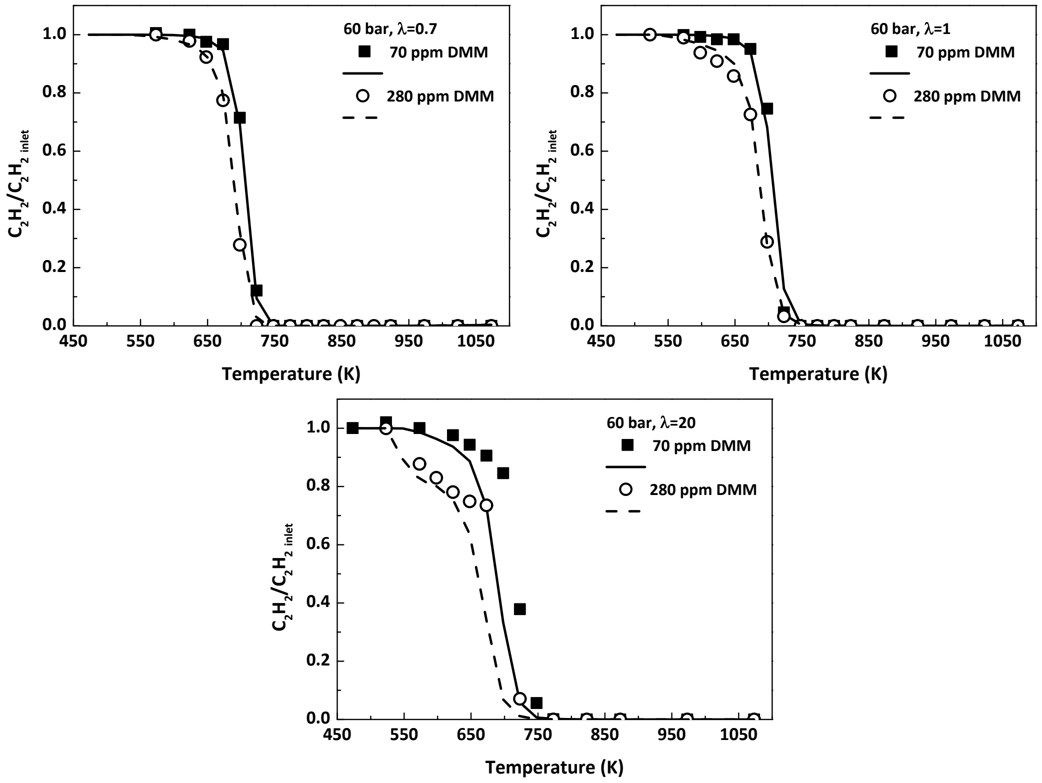

Figure S22. Comparison of the effect of DMM concentration in the reactant mixture (70 or 280 ppm) on the conversion of C_2H_2 for 60 bar and different values of the air excess ratio analyzed.

5. References

[20] Marrodán, L.; Royo, E.; Millera, Á.; Bilbao, R.; Alzueta, M.U. High pressure oxidation of dimethoxymethane. *Energy Fuels* **2015**, *29*, 3507-3517.

[21] Marrodán, L.; Monge, F.; Millera, Á.; Bilbao, R.; Alzueta, M.U. Dimethoxymethane oxidation in a flow reactor. *Combust. Sci. Technol.* **2016**, *188*, 719-729.

[23] Vermeire, F.H.; Carstensen, H.H.; Herbinet, O.; Battin-Leclerc, F.; Marin, G.B.; Van Geem, K. Experimental and modeling study of the pyrolysis and combustion of dimethoxymethane. *Combust. Flame* **2018**, *190*, 270-283.

[26] Li, N.; Sun, W.; Liu, S.; Qin, X.; Zhao, Y.; Wei, Y.; Zhang, Y. A comprehensive experimental and kinetic modeling study of dimethoxymethane combustion. *Combust. Flame* **2021,** *233*, 111583.

[38] ANSYS Chemkin-Pro 17.2; Reaction Design: San Diego, 2016.

[39] Giménez-López, J.; Rasmussen, C.T.; Hashemi, H.; Alzueta, M.U.; Gao, Y.; Marshall, P.; Goldsmith, F.; Glarborg, P. Experimental and kinetic modeling study of C_2H_2 oxidation at high pressure. *Int. J. Chem. Kinet.* **2016**, *48*, 724-738.
